# SSCC TD: A Serial and Simultaneous Configural-Cue Compound Stimuli Representation for Temporal Difference Learning

**DOI:** 10.1371/journal.pone.0102469

**Published:** 2014-07-23

**Authors:** Esther Mondragón, Jonathan Gray, Eduardo Alonso, Charlotte Bonardi, Dómhnall J. Jennings

**Affiliations:** 1 Centre for Computational and Animal Learning Research, St Albans, United Kingdom; 2 Institute for Complex Systems Simulations, University of Southampton, Southampton, United Kingdom; 3 Department of Computer Science, City University London, London, United Kingdom; 4 School of Psychology, University of Nottingham, Nottingham, United Kingdom; 5 Institute of Neuroscience, Newcastle University, Newcastle upon Tyne, United Kingdom; University of Houston, United States of America

## Abstract

This paper presents a novel representational framework for the Temporal Difference (TD) model of learning, which allows the computation of configural stimuli – cumulative compounds of stimuli that generate perceptual emergents known as configural cues. This Simultaneous and Serial Configural-cue Compound Stimuli Temporal Difference model (SSCC TD) can model both simultaneous and serial stimulus compounds, as well as compounds including the experimental context. This modification significantly broadens the range of phenomena which the TD paradigm can explain, and allows it to predict phenomena which traditional TD solutions cannot, particularly effects that depend on compound stimuli functioning as a whole, such as pattern learning and serial structural discriminations, and context-related effects.

## Introduction

Classical conditioning is a fundamental associative paradigm in which repeated co-occurrence of two initially unrelated stimuli results in the acquisition of a new pattern of behavior, commonly assumed to result from the formation of a link (or *association*) between the stimuli's mental representations. Procedurally, it often involves pairing an originally neutral stimulus (e.g., a tone), and a stimulus that is biologically relevant, the unconditioned stimulus (US), or *reinforcer* (+). Once the association is formed, the presentation of the first stimulus (the conditioned stimulus, or CS) comes to activate the representation of the US by means of the link between them. Behaviorally, the CS elicits a conditioned response (CR), indicating that the US is anticipated –effectively predicted by the CS.

One of the most influential models of associative learning, the Rescorla-Wagner model of classical conditioning [1], states that for learning to occur the US must be surprising or, more precisely, unpredicted. Accordingly, the increase in associative strength (*V*), where *V* represents the weight of the CS-US link on a particular CS-US pairing, is proportional to the degree to which the US is unexpected (the delta rule). With each CS-US pairing (trial) the prediction error –the discrepancy between the predicted outcome and the *actual* outcome– is reduced, increasing the associative strength between the elements until the CS fully predicts the US, at which point no further learning occurs. Thus, large prediction errors during early conditioning trials produce large increases in associative strength, but these changes decrease in size as learning progresses and the ability of the CS to predict the US grows, until associative strength approaches asymptotic levels. Formally, 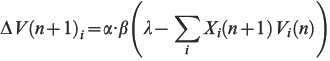.

Temporal Difference (TD) [2]–[3] is considered a real-time extension of the Rescorla-Wagner model. As a real-time error correction model, TD exploits the success of Rescorla-Wagner (for a summary, [4]) and, being able to reproduce timing gradients of response, extends it to explain the pattern of intra-trial acquisition –an advance of significant theoretical importance because it incorporates timing effects within associative theory. Because the prediction error is calculated on each time-step, TD has the potential to deal with some temporal primacy effects (e.g., [5]). Additionally, unlike the Rescorla-Wagner model, TD provides an explicit mechanism to model higher-order conditioning [6] — although it can be argued that the Rescorla-Wagner model explains second-order conditioning by assuming that a CS that has undergone conditioning acts as a standard US.

Notwithstanding its merits and potential, the original formulations of TD are unable to fulfill the requirements of newer research directions. One important weakness of the model lies in the way in which the stimuli are represented: only individual stimuli are instantiated. Relying exclusively on an elemental representation of the stimulus poses serious problems in predicting phenomena that depend on compound stimuli functioning as a whole. This is a significant drawback, because compound stimuli are a cornerstone of many key learning paradigms ranging from simple linear additive effects (e.g., summation test for inhibition) to complex stimulus discrimination procedures (e.g., positive patterning). Processing of compound stimuli has dominated the learning literature in recent years (e.g., [7]–[11]). Hence there is an urgent need to adapt TD to deal with compound stimuli. Indeed, it has been acknowledged that future extensions of TD “will require new formalisms that may attach additional components to the TD model, such as (…) configural representational elements” ([12] pp. 318).

In this paper we present an extension to the Temporal Difference model, the Simultaneous and Serial Configural-cue Compound stimuli Temporal Difference model (SSCC TD henceforth), which incorporates a representation for compound stimuli that includes the notion of configural cue – a kind of perceptual emergent unique to a given combination of elements –which acquires and competes with other cues to obtain associative strength like an orthodox stimulus [13]–[14]. The SSCC TD model allows the representation of stimuli that co-occur simultaneously or in close temporal proximity as a set formed by the individual stimuli and an additional configural-cue stimulus. First, the model posits that a compound representation can be formed between two or more stimuli that coexist simultaneously in time at some point within a conditioning trial, such that their representations are active at the same time. A second, and highly significant feature is that SSCC TD also introduces an algorithm to allow for the formation of a compound representation of serial stimulus compounds for which an active stimulus representation overlaps with the memory traces of earlier, no longer present, stimuli. Compounds can also be formed between the experimental context and a stimulus.

In short, SSCC TD is a computational error correction model of classical conditioning that incorporates an ontology for representing compound stimulus configurations in a real-time architecture using well-established concepts of trial-based associative learning theory. In so doing, SSCC TD overcomes drawbacks of both trial-based and real-time error corrections models: By incorporating a means of representing configurations of stimuli as separate entities, it enables TD to explain performance on the plethora of learning tasks that rely on compound features rather than simple elements, as is the case in most learning paradigms for which stimulus discrimination and generalization are inherent factors. This enables TD to begin to engage with the currently vigorous debate on this issue in learning theory. Moreover, because of TD's intrinsic ability to deal with real-time behavior, these developments in stimulus representation also allow it to be applied to performance on time-based discriminations such as serial-feature and serial structural discriminations.

The paper is structured as follows. First we introduce Temporal Difference learning, and then we present the SSCC TD model formally. Next, we show simulation results that are consistent with standard TD (second-order conditioning, blocking, and timing behavior), then simulations of results that can only be predicted by SSCC TD, namely stimulus generalization, renewal, patterning and biconditional discriminations, summation, feature-negative discriminations, and serial structural discriminations. All simulations are compared against well-known experimental results, showing the predictive power of the new model.

## Model Description

### Temporal Difference

Three distinctive characteristics can be considered as critical in distinguishing between the Rescorla-Wagner model and Temporal Difference.

The first refers to the way in which stimuli are represented. In the Rescorla-Wagner model a stimulus *i* is considered a single entity that is present or not on a given trial *n*, 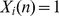 or 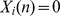, respectively, whereas the most influential representation of TD, the Complete Serial Compound (CSC) representation [15], conceptualizes a stimulus as a temporally distributed set of components that are each effectively treated as distinctive stimuli. Each component is active for only one time-step, and each time-step consists only of a single component. Thus, 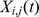 refers to the presence of component *j* of stimulus *i* at time *t*. An example with step by step calculations of how the equations of the model apply and a glossary of symbols and parameters including range of values is presented in Appendix S1.

Second, in TD, an *eligibility trace* is attached to each component which varies in time as a function of two parameters: a decay parameter rho (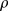), which can be thought of as an index of the component's level of activation (a sort of memory trace), and a discount factor, gamma (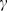), which modulates the eligibility trace according to the component's proximity to the US. A stimulus component's eligibility trace is maximum when the component is present (active) and decays with time. Time is defined in relation to a putative fixed point, the appearance of the US, which is assumed to occur at time 0. Eligibility traces modulate the extent to which the stimulus's associative strength is susceptible to change on any given time-step. Thus, recent stimulus components will have high trace values, allowing for larger changes in associative strength. This means that conditioning will usually be stronger for components close to the US than for components remote from it. In other words, eligibility traces determine the degree of learning that each component can attain according to a component activation value and to its proximity to the US.

Third, instead of calculating the prediction error as the discrepancy between the current trial outcome,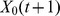, and the predicted outcome,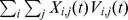 (*simple prediction error*), in TD predicted outcomes are computed at any given time against the outcomes predicted at the next time-step (*temporal-difference error*). Thus, the TD error takes the form
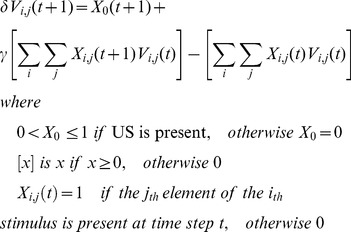
(1)


The temporal difference error entails that, rather than making a prediction based on the cumulative values of all components that are present at time 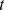 across all stimuli, and then waiting until the outcome is known to update the learning rule, updating is made on the basis of the difference between *successive* predictions. The assumption is that a prediction from the next time-step is based on more recent information and hence is likely to be a better outcome predictor. This is formalized by the operation of a discount factor that results in an exponential decay of the learning update with time, reflecting the fact that predictors closer to the US are more informative and therefore more accurate.

This conceptualization of the prediction error results in a modified delta rule, which is applied once per time-step as opposed to once per trial. In fact, the Rescorla-Wagner delta rule can be treated as a special case of the TD delta rule in which stimuli only last for a single time-step. The associative strength *V* for a component *j* of a stimulus *i* at time-step 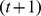 is expressed as:
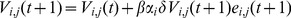
(2)


Equation (2) states a prediction based on the current associative strength, 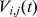, added to the temporal difference error, 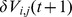, an estimate of how wrong the previous prediction was, based on current information, modulated by a US-dependent learning parameter,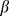, a CS learning rate, 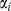, and, crucially,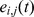, the eligibility trace indicating the extent to which this weight is eligible for modification according to Equation (3), 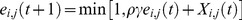
(3)


This eligibility trace is known as a replacing trace – an accumulating trace bounded to a maximum of 1. In summary, in CSC TD stimuli are atomized and each component mapped into a single time unit. As a result, each component has its own associative strength and eligibility trace. It must be noted that in CSC the term “compound” alludes to the temporal representation of a single stimulus, not to the standard associative notion of “compound stimulus” referring to the additive combination of stimuli [16]–[18]. Although other stimulus representations have been proposed in TD (Presence [2], and Microstimuli [12]), CSC has become standard in studies of dopamine function [19]–[25], and is the prevailing interpretation of TD in associative learning studies (e.g., [26]–[28]). Furthermore, the CSC approach is central to the study of reward-based models of schizophrenia (*e.g.*, [29]–[31], for a review).

### Stimulus representation: from CSC to configural-cue compound stimuli

Although the CSC stimulus representation conceptualizes a stimulus as a set of distinctive, temporally distributed components, no other assumption is made about the nature of the stimulus elements *per se* or about how to represent a compound of more than one stimulus. This is a significant shortcoming, as many learning paradigms rely on the representation of stimulus compounds, and on the theoretical assumption that the associative strength of a compound is equal to the sum of its component stimuli.

For instance, the CSC representation does not easily envisage *stimulus generalization*. Standard theories of associative learning, such as the Rescorla-Wagner model, conceptualize a stimulus as being composed of a number of constituent elements (see [32] for an early S-R development of this idea). Each element can enter into association and contribute to the conditioning of the stimulus at any given time. Any two stimuli A and B would have a set of unique elements (e.g., a_1_, a_2_, and b_1_, b_2_, respectively) and a number of elements common to both (e.g., x_1_, x_2_), and the associative strength of the stimulus is equivalent to the sum of the associative strengths of its constituent elements (the *summation* assumption). Generalization occurs by virtue of these shared elements (e.g., [1], [32]–[35]). Consider, for example, Pavlov's original work on discrimination ([36], pp. 121). A dog was presented with two shapes: when the shape was a luminous circle, food was given; when it was a luminous square the dog received no food. One stimulus, the circle for example, can be defined as a compound formed by two elements A and X, A representing its unique features and X those held in common with the square that would, in turn, be represented as being composed of B and X. Thus, during circle-food presentations, both A and X would become associated with food. When the square, BX, is then presented to the animal, the presence of X would engender a CR – that is, stimulus generalization would occur.

A simple solution allowing TD to account for stimulus generalization would be to represent the co-occurrence of multiple stimulus elements at each time-step as constituent entities that could be learned about independently and compete to gain associative strength. Accordingly, the set of elements coexisting in a given time-step could be represented as a stimulus compound, whose associative strength is computed as the sum of the strength of its constituent elements – thus mimicking Rescorla and Wagner's conceptualization of compound stimuli and its summation rule. The Rescorla-Wagner model assumes that the associative strength of a compound stimulus that is being conditioned is the sum of the associative strengths of each constituent stimulus, and stipulates that these stimuli share a limited amount of associative strength up to a maximum value –the US asymptotic level [37]. Conceptualizing the stimulus, and calculating its total compound associative strength, in this manner would enable CSC TD to model standard compound stimuli in the same way as proposed by the Rescorla-Wagner model.

However, the assumption of summation (the idea that the associative strength of a stimulus compound is no more or less than the sum of the strengths of its component elements) is not exempt of problems. Indeed, historically most of the opposition to elementalist accounts originated from demonstrations that responding to a stimulus compound could not be reduced to responding to its individual components (e.g., [38]). Although several elementalist approaches such as Hull's *afferent neural interaction* hypothesis were proposed in response to this criticism [33], the debate remained open. No surprise then that, almost as soon as the Rescorla-Wagner model was proposed, the summation rule was called into question. For example, the successful solution of relatively routine discriminations such as positive patterning was only partially predicted by the Rescorla-Wagner model, while solution of negative patterning was quite impossible.

To solve this problem, it was assumed that two or more stimuli presented together in time are represented as a set of units corresponding to their individual components, plus a configural representational unit that is unique to this stimulus combination and that acquires and loses associative strength by standard associative mechanisms [13], [37], [39]–[41]. As a result, the associative strength of a ‘configural’ compound stimulus can be computed as the additive value of all of the individual and configural units. That is, the assumption of summation is extended to include a configural cue along with the elements of the stimuli comprising the compound [14]. In a negative-patterning discrimination, for instance, two stimuli A and B are reinforced while a compound stimulus AB is not (A+, B+, AB−). The notion of a configural cue permits representation of this discrimination as A+, B+, AB*X*−, where *X* represents the configural cue. The Rescorla-Wagner model would then predict that *X* will become *inhibitory* as opposed to excitatory. Consequently, *X* will counteract the additive effect of A and B on compound trials allowing the discrimination to be solved.

The conceptualization of configural cues thus allowed the Rescorla-Wagner model to successfully predict the correct solutions to non-linear discriminations such as negative patterning, overcoming the summation problem to a certain degree. Since then, there have been different elaborations for representing stimulus compounds, ranging from those in which the elemental approach is abandoned in favor of a pure configural one [9], to those which introduce an elemental point of view that represents stimulus compounds by inhibiting the activation of some of its elements [8] or by replacing some of its constituent elements [42]. In fact, even when not formally incorporated, the idea that the elements activated by a compound stimulus representation differ from those individually activated by each of its constituent stimuli is accepted by almost all associative models. If the TD model is to be used as a realistic means of making predictions about mainstream learning paradigms, then it is essential that we incorporate mechanisms to represent compound stimuli. The approach we have followed postulates added configural cues *à la* Wagner and Rescorla.

We present algorithms that generate configural cues as envisaged by Wagner and Rescorla [14], and a formal representation of configural-cue compound stimuli (that is, compound stimuli that include configural cues). The configural representation is embedded within the well-established CSC TD real time framework. By doing so, the model enhances the explanatory capabilities of Temporal Difference to accurately predict solution of complex discriminations, such as negative patterning, that rely on a stimulus compound structure, while retaining CSC TD temporal characteristics and therefore inheriting its success in predicting a number of temporal related phenomena. To our knowledge the introduction of configural cues has never been considered within the context of TD –perhaps because implementing them in a real-time model is not straightforward. There are several possible approaches. For example, compound stimuli could be defined in terms of the presence of the constituent stimuli on a given trial. Thus we could assume that a compound stimulus is formed if the stimuli are present on the same trial, with independence of the lengths and concurrence of the constituent stimuli. Learning would be effective in real-time for each stimulus –the learning rule being updated at every time unit– but the representation of the compound would be trial-based. An alternative suggestion is that compound stimuli are defined in terms of the real time presence of the stimuli. That is, compound stimuli would be formed only when their constituents overlap in real time and only for the duration of the overlap. In this case, both representation and learning are computed in real time. We have adopted the latter approach.

Next, we present the formal representation of the SSCC TD model for simultaneous and serial compound stimuli. To avoid confusion with the CSC terminology of TD we will henceforth refer to a compound of stimuli that includes a configural cue as a stimulus *configuration*. Thus, a configuration consists of a set of *configuration constituents*, which include the stimuli themselves, called *primitive constituents*, and the corresponding emerging *configural cue*. Stimuli, configural cues and configurations are comprised of a number of CSC *components*. To illustrate our terminology: consider a compound of two stimuli, A and B, which co-occur at a given time-step; A and B are the primitive constituents at that particular time-step; 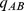, is the emergent configural cue; A, B, and 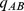 are the configuration constituents of the configuration 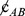.

#### Simultaneous Configurations

**Figure 1:**
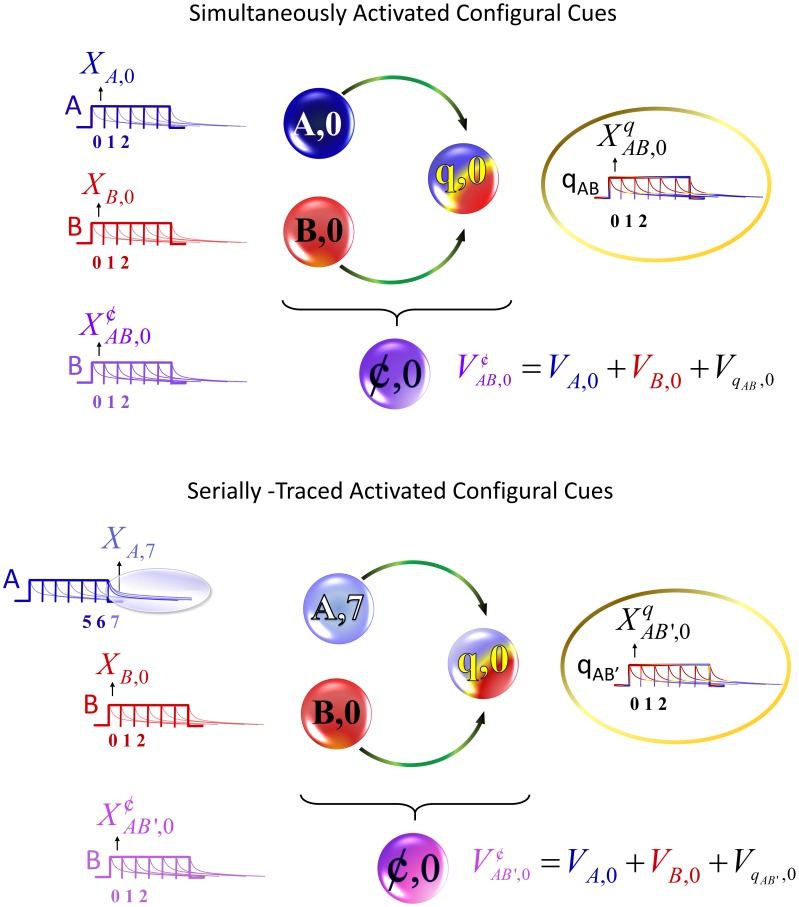
Configural-cue representation for simultaneous and serial configurations. Schematic representation of the formation of a configuration 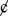 from stimuli A and B and its associative strength. The top panel depicts simultaneous activation of the stimuli (their presence, 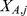 and 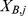) and the emergence of the corresponding configural cue q (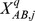); the bottom panel represents its serial counterpart where traces of stimuli are involved. The formula to estimate their respective associative strengths (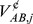 and 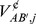) is also depicted.

Whenever two or more stimuli co-occur, a *simultaneous* configuration is formed. Such configurations are represented as a unit composed of all the primitive constituent stimuli plus a configural cue. The associative strength of a configuration is then calculated as the arithmetic sum of the value of the configuration constituents. Thus, in order to compute the configuration's associative strength, we need first to formally define the conditions for the presence of the configuration (to distinguish presence of a configuration from presence of a stimulus, 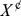 is used instead of 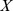). Notice that establishing the presence of the primitive constituents (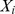) of a configuration is enough to verify the presence of the configuration itself. That is, we need to computationally describe a way to represent the co-occurrence of the primitive constituents. Once the co-occurrence of the configuration is established, the presence of the corresponding configural cue is instantiated, with a presence denoted by 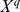. Next the SSCC TD learning algorithm computes the associative strength of the constituents of the configuration. These values are summed to give the configuration strength. A schematic representation of this process is shown in the top panel of Figure 1.

*Configuration representation*. Stimulus configurations are assumed to exist when there is a temporal overlap between their primitive constituents. In the SSCC TD model, the process of creating a configuration is performed on each time-step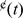. The presence of a configuration *m* at a given time *t*,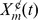, is determined as a function of the presence of the primitive constituents, such that 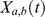 represents the presence of the 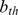 CSC component of the 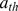 primitive constituent at time 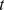. Thus, the presence value for a configuration is calculated as the product of the sums of the vectors for all components of all the stimuli. In addition, a configuration is present only if exactly all its primitive constituents are present, such that a configuration AB is not present if stimuli A, B and C are present; configuration ABC will be present instead. This condition is made explicit in the subtractive term in Equation (4), where 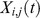 represents the presence of the 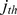 component of the 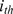 stimulus excluding the configuration and its primitive constituents at time 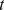. Formally,
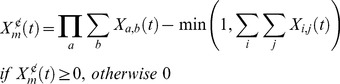
(4)


Equation (4) gives us a time-transversal binary representation (i.e., a snapshot at a given time *t*) of the presence of a configuration. If present, Equation (4) outputs a value of 1, otherwise 0. In other words, a compound is present if Equation (4) equals 1, absent otherwise. For example, consider the case in which two stimuli A and B co-occur at time *t*. The presence of the configuration 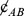 (*m* = AB) at *t* will be determined by multiplying the presence values of all the components *n* of the two stimuli that are or have been active until that point minus the minimum value between 1 and the sum of the presence of any other stimulus component at *t*. If the current components are the initial components (that is, if 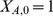 and 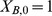), the product of the sums in the first term of Equation (4) reduces to the product of the presence values of A and B. In addition, if there are no other stimuli present at that time, the second term outputs zero. That is, 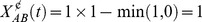. As a result, the presence of the configuration AB is established at *t*. Assume now that a third stimulus C is present and that A, B, and C co-occur at *t* and we ask whether the configuration AB is formed. Equation (4) would output 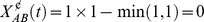. That is, the presence of AB is rejected, whereas the presence of the configuration ABC is confirmed: 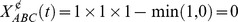. The CSC representation assumes that there is a direct mapping between a time-step and a component, Thus, if A and B remain present at *t*+1 their respective initial components will become inactive (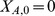 and 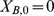) and the next components in the sequence will become active (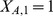.and 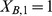). Now the presence of the configuration 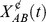
will be estimated by multiplying the sum the component presences of A 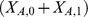 and the sum of the component presences of B 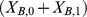 and subtracting from this value the minimum between 1 and sum presences of any other stimulus component at *t*+1. That is, 
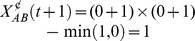
. A step-by-step example illustrating the computation of stimulus presences and their associative strengths is given in Appendix S1.

In order to make Equation (4) operational in real time we need to specify when the configuration will be initialized and how its presence will be calculated subsequently. In Equation (5), 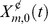 defines the presence of the *first* component of the configuration in terms of change in the presence of the configuration components from time 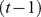 to 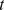. That is, when the difference between the configuration component presence at 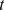 and the configuration component presence at 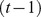 as given in Equation (4) equals 1, then the presence of the first component is initiated. In other words, a configuration component *m* is the *first* configuration component at time *t* when it becomes present in *t* (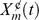 = 1) and the previous configuration component *m* is absent (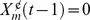).
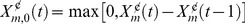
(5)


Finally, in Equation (6) 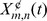 gives the presence of the 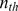 component of the 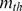 configuration at time 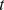. Since only a single CSC component of a stimulus can be active at any time-step, this yields 1 only if all stimuli have a present component, and otherwise yields 0; as such, the presence of the 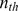 component is contingent on the immediately preceding component.
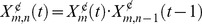
(6)


*Configural cue representation*. The presence of a configuration 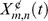 entails the existence of a configural cue, 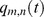. Thus, Equation (7) establishes the presence of the configural cue as follows: 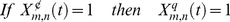
(7)


This configural cue is represented as a new stimulus *i* that becomes an extra configuration constituent. Configural cues denote a unique feature that results from the perceptual combination of the primitive constituents, and as such it operates at a sensory level without the intervention of an explicit learning mechanism. While in the Rescorla-Wagner model configural cues are formed when more than one stimulus is present in a trial, in the real-time world of SSCC TD we assume instead that these configural cues exist only when the CSC components of their primitive constituents overlap, and during the time they overlap. In all other respects they are treated as any other stimulus configuration constituent.

**Figure 2:**
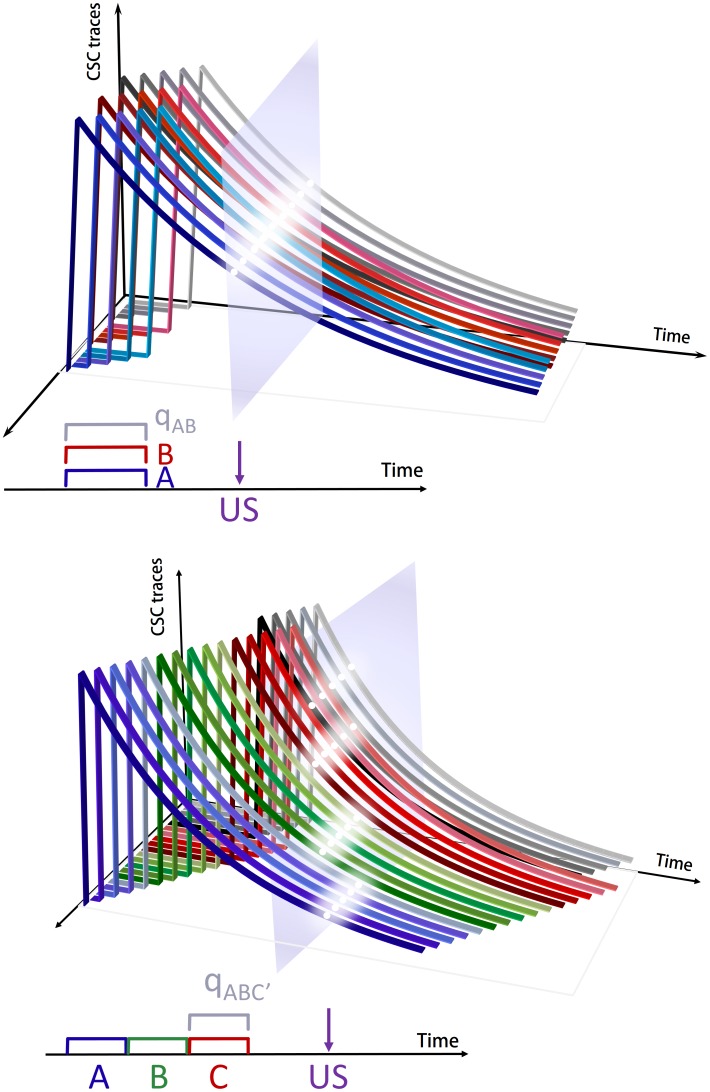
Eligibility traces for simultaneous and serial configurations. Top panel: Eligibility traces of two 5 s simultaneously presented stimuli A and B, and of the resulting configural cue q across time, and their intersection with the US onset, following a stimulus trace interval. Bottom panel: Eligibility traces of three 5 s serially presented stimuli A, B, and C, and of the resulting configural cue q across time, and their intersection with the US onset, following a stimulus trace interval.

*Constituents learning rule*. All configuration constituents, including the resulting configural cue, compete against each other for the available associative strength following Equation (1). The TD error is then modulated as in Equation (2) by the eligibility traces. The top panel of Figure 2 illustrates the constituents' eligibility traces for a configuration AB, formed by two stimuli A and B that co-occur simultaneously along five temporal units before the US presentation.

*Configuration associative strength*. The associative strength of a configuration *m*, (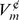), is then determined by the sum of the strengths of its constituents rather than directly by the repeated application of the error prediction algorithm –configurations are not learned about, only their constituent stimuli, which include the additional configural cue. 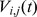 refers therefore to both primitive constituents and configural cues. Hence SSCC TD prescribes the following modified equation for 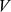: 
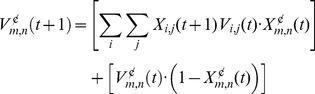
(8)


The first term of Equation (8) computes the sum of the associative strength of all configuration constituents. The second term returns the configuration associative strength of the previous component if the configuration presence ceases, that is when 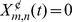; otherwise, when 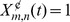, the second term vanishes and thus only the first term is in effect.

#### Context representation and context-stimulus configuration

In many cases the configurations consist of discrete stimuli - but in others one constituent is in fact the experimental context. Although the context may be thought as a collection of elements, in this paper the context is instantiated as a single stimulus – as it is *de facto* abstraction in most learning models – acting as a primitive constituent of a configuration. Incorporating contextual cues as constituents will enable the SSCC TD model to successfully predict a number of basic phenomena such as the systematic effect on associative strength of variations in the contingency between CS and US. Treating the context in this way also makes it possible to model complex contextual procedures such as context blocking in fear conditioning (e.g., [43]) and renewal after extinction effects (e.g., [44]).

There is no explicit consideration of how the context is modeled with a CSC stimulus representation. SSCC TD represents a context as a distinct stimulus, which lasts for the duration of the trial and repeats immediately after the trial ends. Equation (9) shows how the duration is derived, with the repetition representing a modification of the stimulus presence in Equation (1) where the components of the stimulus are advanced through with respect to modulo 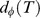.



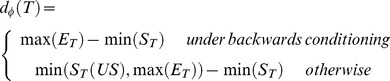
(9)where 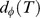 is the duration of the 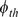 context in trial 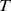, 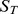 is the set of start times for stimuli in trial 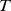, 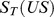
is the onset time of the US in trial 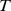, and 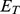 is the set of offset times for stimuli in trial 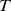. This operation results in a stimulus that begins at time-step zero and then repeats constantly throughout the duration of the trial, including the inter-trial-interval (ITI). This allows the contextual stimulus to lose associative strength during the ITI as it repeatedly occurs in the absence of the US. Aside from their repeating nature and the method used to calculate their duration, contexts are treated in exactly the same way as other stimuli and thus are capable of contributing to configurations and hence to configural cues.

#### Serial Stimulus Configurations

A further and perhaps more interesting development is proposed in this paper. Incorporating configural units that contribute to the formation of configurations in temporal difference learning considerably enhances the ability of the model to cope with a number of non-linear discriminations. However, so far we have only considered configurations that are formed when the stimuli are concurrent. Although this is the case for most of the complex discriminations mentioned above, other crucial phenomena in associative learning theory depend on stimuli presented sequentially rather than simultaneously. In serial feature discriminations, for example, differential response depends on different configurations of stimuli that are serially presented. Thus, in a serial feature-positive discrimination, a target stimulus T is reinforced only when preceded by another stimulus F (for feature); a serial feature-negative discrimination procedure involves the reverse contingency, that is, an otherwise reinforced target stimulus is not reinforced when signaled by F. Moreover, being able to represent serial stimulus configurations in a real-time model allows us to deal with learning phenomena that, at face value, seem to lie beyond the scope of an associative interpretation. Up until this point, in all the procedures considered, discrimination is based on the presence of differential elements in each type of reinforced condition. In other words the set of elements that compose the reinforced configuration is never entirely the same as the set included in the non-reinforced configuration. A further increase in discrimination complexity comes from procedures for which reinforced and non-reinforced cues differ solely on the basis of how the constituent elements are ordered. Pavlov [36] described what can be considered the simplest form of serial pattern discriminations in which the same set of stimuli, A and B, are both reinforced and not reinforced. When A precedes B, food follows but if B appears before A, food is omitted. Pavlov's serial pattern discrimination can easily be dealt with by standard associative theories by appealing to the differential associative strength of the elements; however, when the associative strength of the two components is well-controlled, serial pattern discriminations [45]–[47] are examples of *serial structural* discriminations in which discriminative performance cannot in principle be acquired by differentiating the sensorial features of the elements involved, meaning its explanation lies beyond the scope of current associative analysis. Instead, discrimination in this kind of procedure seems to entail distinguishing non-modal stimulus properties, such as the order of presentation.

One way to approach this problem is to assume that for every serial configuration a unique cue is formed, a cue that is specific to the particular order in which the stimuli occur. Thus, in addition to representing a perceptual emergent of the sensory properties of the stimuli, serial configural cues would include information on how these stimuli are mapped in time. We assume that when two stimuli are presented contiguous in time a configuration may be formed. As in the case of simultaneous stimuli, the associative strength of this configuration would be computed as a summation of the associative strength of its primitive constituents and of their specific configural cue.

The notion that serial stimulus compounds could result in configural learning has previously been acknowledged within the elementalist framework (e.g., [48]–[49]). Sutherland and Rudy [50] postulated that configural representations involve a controller cue formed by elemental stimuli that occur either simultaneously, sequentially or distributed in a given spatial relationship. According to this proposal, elemental and configural associations rest on different sets of learning and memory systems that share a number of neural components but differ in the involvement of the hippocampal formation: whereas configural associations would critically depend on the hippocampal formation, elemental associations would not. To our knowledge, however, no real-time *output* mechanism has been proposed to account for the formation of configurations for sequential stimuli (but see the discussion section). We present here a method to generate serial stimulus configurations.

*Serial configuration representation*. The temporal mechanism of CSC TD may be used to build a formalization for serial configurations. In CSC TD each component is fully and sequentially activated in time. Each activation slowly decreases according to a decay trace that lasts long beyond the stimulus offset and thus can effectively be taken as the CSC memory trace. Thus, for instance, the CSC eligibility traces of a stimulus A, that precedes another stimulus B, will coexist in time with active CSC components of B. In SSCC TD we assume that the memory traces of A interact with the active traces of B to generate a configural cue and contribute to the formation of a serial stimulus configuration A→B. A schematic of this interaction is shown at the bottom panel of Figure 1.

The computational representation of serial configurations requires distinguishing between eligibility traces of the stimulus that is currently present and the traces of constituent stimuli that have already ended. The bottom panel of Figure 2 depicts the CSC eligibility traces for three serially presented stimuli A, B and C. The associative strength of a serial configuration is calculated during the final primitive constituent, C, when the traces of the preceding stimuli, A and B, co-occur with the active traces of C. As with simultaneous configurations we are establishing the condition that a configuration is present if and only if all its primitive constituents are present within a trial; therefore a configuration AB is not present if stimuli A, B and C or their traces are present –an ABC configuration is formed instead. Obviously, which set of stimuli comprises a specific serial configuration in a given trial is predefined by the experimental design.

Formally, the presence of any given component of a serial configuration is binary coded by Equation (10) in which 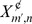 represents the presence of the 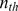 component of the 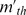 serial configuration formed by *z* stimuli at time *t*, where 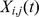 denotes the presence of the 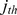 component of the 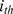 stimulus at time *t*, and 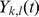 is the presence of the trace of the 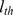 component of the 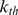 stimulus. The *signum* function (sgn) could also yield a -1 value if negative components were to exist, but this is clearly not a defined case.
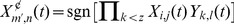
(10)


If a configuration component 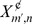
is present, that is, if 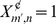, a configural cue 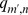 is added as a new stimulus *i* that will input Equation (1). Notice that we use a different notation for the presence of stimuli and their components (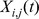) and for the presence of traces and their components (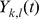). We also use primes to distinguish between simultaneous and serial configurations. Nonetheless, the main concept is intuitive and simple, that for a serial configuration to be present there must be at least one stimulus present, that is, that serial configurations are not formed by traces only. This is ensured by condition *k*<*z*, where *z* is the total number of stimuli and *k* refers to the stimuli that are traced at *t* but not currently present at *t*. In other words, serial configurations are formed during the time in which stimulus traces overlap with at least one active stimulus, with exclusion of the context. The associative value of the context during this time, however, does contribute to the configuration's value.

The associative strength 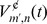 of the 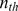 component of the 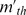 configuration of stimuli at time *t* is then calculated as follows:
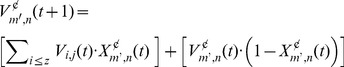
(11)


Unlike simultaneous configurations, in which the configuration is formed while all stimulus representations are active, serial configurations are formed once the activation of the representation of the earlier stimuli has decayed and the activation of the last stimulus representation is at its maximum. Thus we can assume that response recorded during the last stimulus of the series in a serial configuration would be determined not only by the associative strength of the stimulus configuration, as with simultaneous configurations, but would also be controlled by the associative strength of the final and fully active individual stimulus. A decision response rule could be used to adjust for these unequal stimulus activation values by applying a weighted arithmetic mean to the predicted response to the last stimulus of the series and the predicted response to the serial stimulus configuration. However, for the sake of parsimony, we have used a single identical response decision rule for stimuli, simultaneous and serial configurations, namely that proposed by Church & Kirkpatrick [27], 
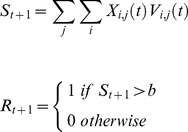
(12)


where *b* is a random number uniformly distributed between 0 and a given threshold *B*. The simulated responses are produced by repeating equation 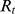 with a new 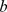 at each iteration and summing the results. Thus, there is a proportional relationship between the calculated responses and the associative strength, and the response rule introduces some variability to the data.

## Results

In what follows we present a series of simulations of prototypical experimental results that exemplify fundamental and well-established classical conditioning phenomena as well as new phenomena for which the SSCC TD representational architecture, with simultaneous and serial stimulus configurations and a real time framework, is required; thus, highlighting the capabilities of the SSCC TD model. These are presented in four blocks. In the first we include experiments – second-order conditioning, blocking and timing– that traditional TD accounts for without assuming configurations. We have added them for the sake of completeness and to show that the new model not only inherits its successes from TD but by using stimulus configurations and context-stimulus configurations to simulate these phenomena, adds resemblance to real experimental conditions.

The second block of results refers to phenomena the model of which requires the explicit introduction of configurations – and thus that traditional TD cannot predict. They include stimulus generalization, renewal effects and conditional non-linear discriminations.

The third block reports an experiment on stimulus summation [51] that aims to demonstrate that the incorporation of configural cues, and thus the configural-cue compounds described in the SSCC TD model, preserve an elemental approach to learning and the summation assumption.

Finally, results of two experiments on serial *vs.* simultaneous negative occasion setting and a serial structural discrimination are simulated. In both experiments, time is a critical variable required to solve the discriminations. It should be stressed that the concept of time refers not only to the idea of *duration* – the main focus of so called timing theories – but also, and essential in classical conditioning studies such as those mentioned above, to the notion of *succession* that indicates that stimuli are perceived differently depending on their order in a given sequence [52]. This last block of results is of paramount importance in that it shows that SSCC TD can potentially predict a range of phenomena, explanations of which have traditionally been considered beyond the scope of standard associative theories.

Simulations were run with a set of fixed parameters except for the α values, which were adjusted to match the empirical learning rates in each experiment as closely as possible. In the timing experiment higher 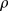 and 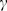 values were used to reduce the slope of the stimulus temporal discrimination. The learning rates for the configural cues were calculated as follows: the product of the two highest α values was used as the simultaneous configural cue rate; the same rule was applied to calculate the serial configural cue rate, but to gauge for memory interference this value was adjusted by a factor calculated as the number of configuration-unique CSs (if bigger than 0, otherwise 1) over the number of configurations that shared one or more CSs with the target configuration (if bigger than 0, otherwise 1). In all simulations in which two or more stimuli were involved, (e.g., A and B) a common element (e.g., X) was assumed. Thus, the simulation represented the nominal stimuli (A and B) as a compound formed by common and unique elements AX and BX.

The design and parameters used in each experiment simulation are presented in Table 1.

**Table 1:** Designs and parameters of the experiments simulated with SSCC TD.

						*Parameters*
						λ = 1; ρ = 0.97; γ = 0.97
	*Group*	*Phase 1*	*Phase 2*	*Phase 3*	*Phase 4*	β+ = 0.5; β– = 0.495
						Decision Rule = 0.985
						Timestep = 1 s
**Second-Order Cond.** *Holland & Rescorla (1975)*	PP	84 L+	16 CL–			α(CSs) = 0.1
	UP	84 L; 84+	16 CL–	-----	-----	α(x) = α(***A***) = 0.001 (1%)
	PU	84 L+	16 C–; L–			
**Blocking** *Allen et al. (2002)*	Blocking	700 T+	500 TL+	30 L–	500 L+	α(CSs) = 0.025
	Control	------	500 TL+	30 L–	500 L+	α(x) = α(***A***) = 0.00025 (1%)
	Naïve	------	-----	-----	500 L+	**Timestep = 0.05 s**
**Timing** *Jennings et al. (2013)*	Group 60	90 C*(f)*+; 90 N*(v)*+;				**ρ = γ = 0.989**
		45 T*(f)* –; 45 T*(v)* –;				α(CSs) = 0.125
			-----	-----	-----	α(x) = 0.00065 (.5%)
	Group 30	90 C*(f)*+; 90 N*(v)*+;				α(***A***) = 0.001 (1%)
		45 T*(f)* –; 45 T*(v)* –;				
**Generalization** *Pavlov (1927)*						α(CSs) = 0.25
		33 A+; 33 B–	------	------	------	α(x) = 0.2 (80%)
						α(***A***) = 0.003 (1%)
**Renewal** *Bouton & Peck (1989)*	ABA	***A*** 40 T+	***B*** 28 T–	***A*** 24 T–		α(CS) = 0.25
	AAA	***A*** 40 T+	***A*** 28 T–	***A*** 24 T–	-----	α(***A***) = α(***B***) = 0.0375 (15%)
	Control	***A*** 40 T; 40+	***A*** 28 T– (***B*** 28 T–)	***A*** 24 T–		
**Patterning & Biconditional Discriminations** *Harris et al. (2008)*	Pos. & Neg.	800 AB+; 400 A–; 400 B–;				
	Patterning	800 CD–; 400 C+; 400 D+				α(CSs) = 0.1
			-----	-----	-----	α(x) = 0.02 (20%)
	Biconditional	800 AB+; 800 CD+;				α(***A***) = 0.001 (1%)
	Discrimination	800 AC–; 800 BD–;				
**Summation** *Rescorla (1997)*						α(CSs) = 0.1;
		176 AB–; 176 AD+; 176 BC+	8 AB–; 8 AD+;	16 AB–; 16 AD+;	8 AB–; 8 AD+; 8 BC+;	α(x) = 0.02 (20%)
			8 BC+; 4 CD–	16 BC+	2 CD–; C–; D–	α(***A***) = 0.001 (1%)
						
**Negative Occasion** **Setting** *Holland (1984)*	Sim	4 N+	12 N+; 36 LN–	8 L+		**ρ = γ = 0.995**
	Sim-C	4 N+	12 N+; 36 L–	8 L+	N–; LN–; LN–; N–;	α(CSs) = 0.495
	Ser	4 N+	48 N+; 144 LN–	8 L+	LN–; N–; N–; LN–	α(x) = 0.055 (10%)
	Ser-C	4 N+	48 N+; 144 L–	8 L+		α(***A***) = 0.0055 (1%)
**Serial Order** **Discrimination** *Murphy et al. (2004)*		210 A→B+; 210 B→C+;				α(CSs) = 0.1
		210 C→D+; 210 D→A+;	-----	-----	-----	α(x) = 0.02 (20%)
		210 B→A–; 210 C→B–;				α(***A***) = 0.001 (1%)
		210 D→C–; 210 A→D–				

*Note*. A, B, C, D, L, N, and T represent different stimuli; ***A*** and ***B*** represent two distinctive contexts; (*f*) indicates fixed stimulus duration; (*v*) indicates variable stimulus duration; + indicates reinforcement; – indicates non-reinforcement; separates different types of trials; → indicates serial stimulus presentation; α, β, γ, λ and ρ represent the corresponding model parameters. Where two or more stimuli appeared within an experiment, a common element x was simulated. Thus, α(CSs) describes the rate of conditioning parameter for the unique CS elements, α(x) the rate of conditioning parameter of the common x element, and α(***A***) and α(***B***) the rate of conditioning parameters used for the contexts.

As noted above, Church and Kirkpatrick's [27] decision response rule was used for the simulations. Results are given as response rates per minute, bounded at a maximum of 100 rpm.

Simulations were run with the SSCC TD Simulator, a universal design-input software that implements the SSCC TD model. The files required to replicate the results, Design Datasets S1, can be downloaded from the Supporting Information section. These files can be opened and run with the SSCC TD Simulator, available at http://www.cal-r.org/index.php?id=SSCC-TD-sim. A Simulator Quick Guide S1 is also available.

The predictions of behavioral models of classical conditioning are mainly concerned with the direction of patterns of behavior. Following standard practice in the field (e.g., [53]–[56]) simulated results were compared against published experimental data by visual inspection of their respective CR patterns.

### Experiment 1. Second-order conditioning

Second-order conditioning is an instance of higher-order conditioning in which a neutral CS acquires associative strength through being paired with a stimulus that has previously been conditioned to a US (e.g., [57]–[59]). The Rescorla-Wagner model cannot, in principle, explain this phenomenon because it does not define a mechanism for associative transfer through successive stimuli; instead learning is driven by the difference between a predicted and an actual outcome (but see footnote 1). Temporal Difference, however, describes learning as a function of the difference between successive predictions of future outcomes and can, therefore, predict second-order conditioning.

In this test, we simulated the results of a study on second-order conditioning reported by Holland and Rescorla [57] Experiment 1. Table 1 shows the design used.

The experiment consisted of two phases. During Phase 1, first-order conditioning to a light was established in two groups, Group PP and Group PU. The light was 12 s long and simultaneous conditioning with an inter-stimulus-interval (ISI) of 10 s was employed. The US consisted of two food pellets delivered over a period of 2 s. A third group, Group UP, received unpaired presentations of the light and the US. Each stimulus was presented 84 times. Phase 2 followed and consisted of 16 presentations of a click and 16 presentations of the light. The stimuli in this phase lasted 10 s and in Groups PP and UP were sequentially presented with an ISI between the light and the click of 10 s. In Group PU their presentations were unpaired. The same design parameters, with an ITI of 665 s, were used in the simulation.

**Figure 3:**
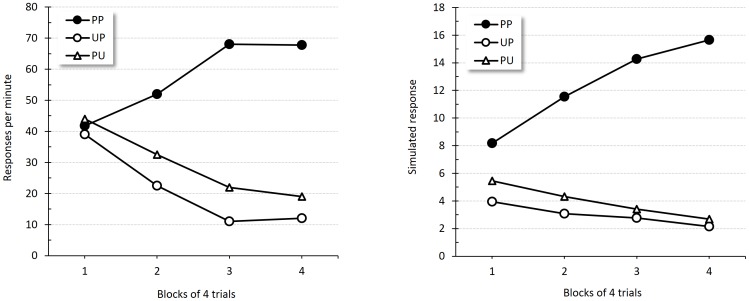
Second-order conditioning. Empirical (original measurement units) and simulated results during second-order conditioning test. Left panel reproduces the group mean response rates to the click across 4-trial blocks during test, adapted from Holland and Rescorla's Experiment 1 [57]. The right panel shows the corresponding simulated responses per minute.

Figure 3 shows responding to the click during Phase 2 of second-order conditioning. Empirical results from Holland and Rescorla's experiment are displayed in the left panel; the right panel depicts the corresponding simulated results. As in their experiment, simulated responding in Group PP increased over time relative to that in Group PU and Group UP. Additionally, no differences between the latter groups were predicted.

### Experiment 2. Blocking in eye-blink conditioning

A simulation of the results of a blocking experiment published by Allen, Padilla and Gluck [60] was performed next. They conducted a simple study to test whether blocking in rabbit eye-blink conditioning is the result of a learned inattention mechanism [61], often mapped to the hippocampus [62], or modulated by an error correction process [1], considered to be mapped to the cerebellum (e.g., [63]).

According to the authors, if blocking is the result of learned inattention then conditioning to a previously blocked stimulus should be slower than conditioning to a novel stimulus, whereas if blocking is the result of an US error correction mechanism, conditioning should develop at the same rate as to a novel stimulus.

Unlike most conditioning procedures, eye-blink conditioning uses very short stimulus durations, often in the range of milliseconds; thus using a time-step length in a similar range is necessary. The design and parameters used for this simulation are presented in Table 1.

A time-step length of 0.05 s, equal to the US duration in Allen *et al.*'s paper, and a variable ITI (30 s±5 s) were used. For animals in Group Blocking, Phase 1 consisted of 700 conditioning trials to a 0.45 s tone, (T+); Phase 2 comprised 500 simultaneous presentations of a light and the tone (TL+), and Phase 3 30 L extinction test trials. Finally, in Phase 4, L was conditioned to the US across 500 trials. Group Control received identical training to Group Blocking except in Phase 1, in which it did not receive any stimulus. Group Naïve only received training during Phase 4.

**Figure 4:**
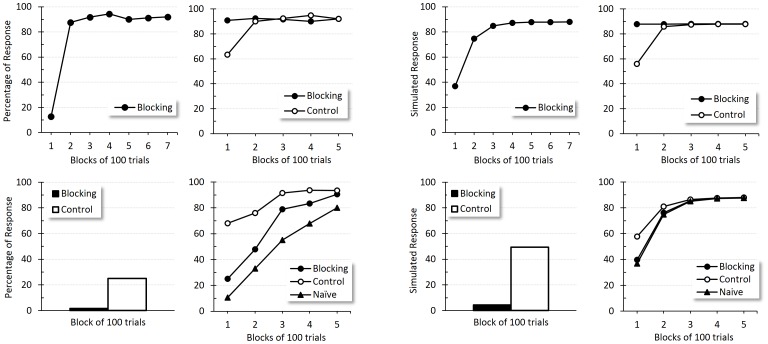
Blocking. Empirical (original measurement units) and simulated results for the four phases of blocking in eye-blink conditioning. Left panel reproduces an adaptation of Allen *et al.*'s results [60]. From left to right and top to bottom: Percentage of response to the tone during acquisition, to the tone-light compound during compound conditioning, to the light during the blocking test and during reacquisition for groups Blocking, Control and Naïve. Right panel shows the corresponding simulated response per phase and group.

Figure 4 shows the results for this experiment. The left panel displays Allen *et al*.'s group mean percentage of response across training and the right panel displays simulated responses. Within each panel, consecutive experimental phases are depicted from left to right and top to bottom. In Phase 1, conditioning to the tone develops unremarkably (top left panel). Phase 2 (top right panel) correctly indicates that the compound TL progressively acquired associative strength in Group Control, while Group Blocking displayed a high and asymptotic level through the phase. Blocking test results (bottom left panel) accurately showed lower conditioning levels to L in Group Blocking than in Group Control. Finally, the rates of conditioning revealed in Phase 4 also matched those of Allen *et al.*'s experiment. Acquisition did not differ in Group Blocking and Group Naïve, but did develop more slowly than in Group Control, confirming that blocking in eye-blink conditioning seems to be better predicted by error correction models such as TD.

### Experiment 3. Timing

Many of the timing models that can explain conditioning differ from associative theories in positing that the rate or level of conditioning is determined by the cumulative duration of the CS, and of the ITI, over a series of trials; the characteristics of individual trials do not necessarily affect the course of learning. In order to test this hypothesis Jennings *et al.* [64] compared learning to a fixed duration CS to learning about a stimulus whose duration varied from trial to trial, but whose mean duration was matched to that of the fixed stimulus.

The experiment employed a within-subjects design, in which all animals received training with two reinforced cues, a fixed duration CS F, and a CS V whose duration on each trial was drawn from an exponential distribution with the same mean as the fixed CS duration. F and V were a click and a noise, counterbalanced. Animals also received non-reinforced presentations of a control stimulus C (a tone), whose duration was fixed on half its presentations and variable on the remainder. For half the animals the mean CS duration was 30 s and the ITI comprised a fixed 60 s plus a variable 30 s interval; in the remainder the mean CS duration was 60 s and the ITI a fixed 90 s plus a variable 60 s interval. There were five sessions of training, each comprising 54 trials, 18 with each type of CS.

Although the simulation added common elements (e.g., X) to the nominal (e.g. tone) stimuli as in previous experiments, in this experiment common elements were not computed as part of the configuration. In the experiment, the stimulus durations varied from trial to trial resulting in different lengths for the common and the nominal elements on each individual trial. Durations were drawn from an exponential random distribution with the same mean for both common and nominal elements, but the specific distributions of common and nominal elements, differed at a given trial. Thus, to avoid the contribution of redundant strength to the configuration that would result from the addition of the associative strength of the common elements during the times at which their lengths differed from the lengths of the nominal elements, data in the figures did not compute the common elements as part of the configuration.

**Figure 5:**
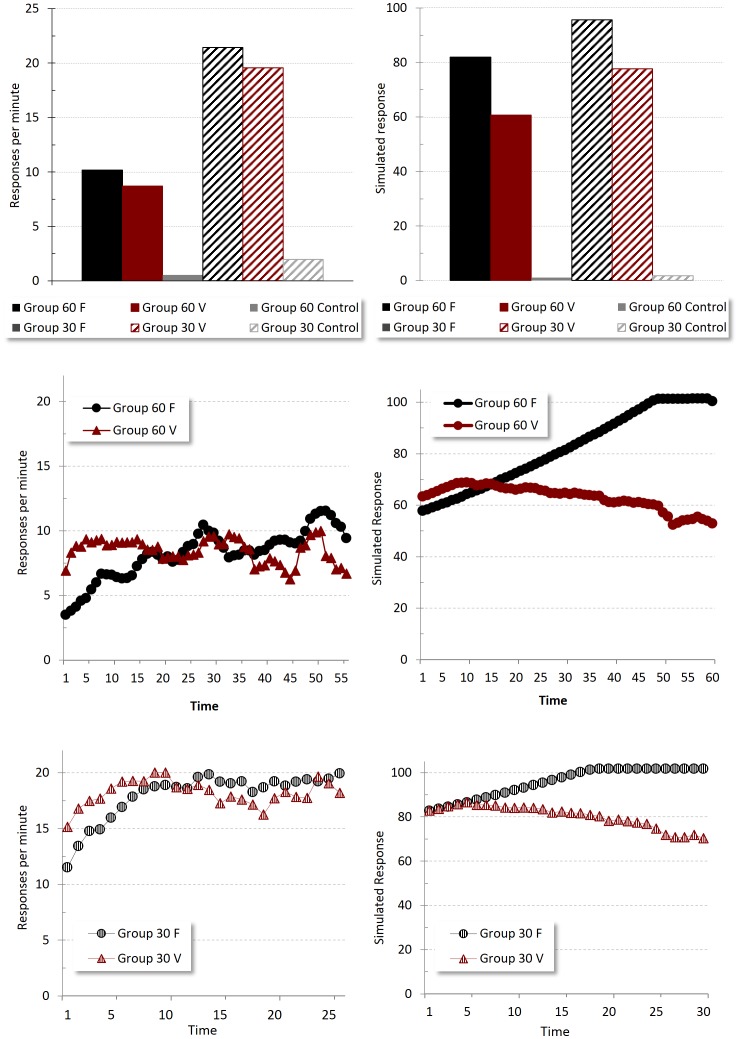
Timing. Empirical (original measurement units) and simulated results of Jennings *et al*.'s Experiment 2 [64]. The top panel shows responding –difference scores per minute- to the three CSs in Group 60 and Group 30 in the final training session. The top-right panel reproduces an adaptation of the empirical results, the top-left panel the corresponding simulated results. The center and bottom panels display responding across time for the 60 s CS (middle panel) and 30 s CS (bottom panel); the left panels show the empirical results, the right panels the simulated corresponding response rate.

Figure 5 shows the results for this experiment. The top left panel shows a measure of responding to the three types of CS in the final training session; the data are presented as elevation scores – the mean rates of responding during each kind of CS after subtraction of the mean response rate during the preCS period (the portion of the ITI that immediately preceded each CS presentation). This gives a measure of the extent to which CS can elevate responding over background levels. For both 60 s (left) and 30 s (right) CSs responding to the reinforced F and V was higher than to the non-reinforced control cue C, but also - critically - responding to the fixed duration CS was higher than to its variable counterpart; in addition, as would be expected, responding to the 30 s CSs was higher than responding to the 60 s CSs. The same general pattern was also evident in the simulation (top right panel), the only marked difference being that rates of responding to both 60 s CSs were slightly higher than in the actual data.

The middle and bottom left panels of Figure 5 show the levels of responding over the course of the reinforced CSs at the end of training, allowing evaluation of the extent to which the fixed and variable duration cues controlled differential behavior patterns. It was anticipated that when the CS was of a fixed duration the animals would be able to time the occurrence of US delivery, which would be reflected as increasing levels of responding as the end of the CS approached - for a fixed CS, the more time elapses the closer the US occurrence. In contrast, elapsing time during the variable CS does not give any information about US proximity, and so for this CS steady levels of responding over the CS's duration were anticipated. The data presented are the group mean corrected response rates for each second of the 60 s CS (middle left panel) and 30 s CS (bottom left panel), smoothed using a 5-s running mean to minimize noise. The general pattern in the data is similar to that in the simulation: crucially, responding to the fixed CSs increases steadily with time, whereas responding to the variable CSs maintains a steady, or slightly downward trend, as the CS elapses.

### Experiment 4. Stimulus generalization

In order to demonstrate how the proposed stimulus representation accounts for stimulus generalization, we simulated the example used in the introduction [36]. Pavlov trained a dog to discriminate two luminous shapes. The presentation of one shape, the circle, was followed by food; the presentation of the other, the square, was not. The design and parameters of the experiment are shown in Table 1.

**Figure 6:**
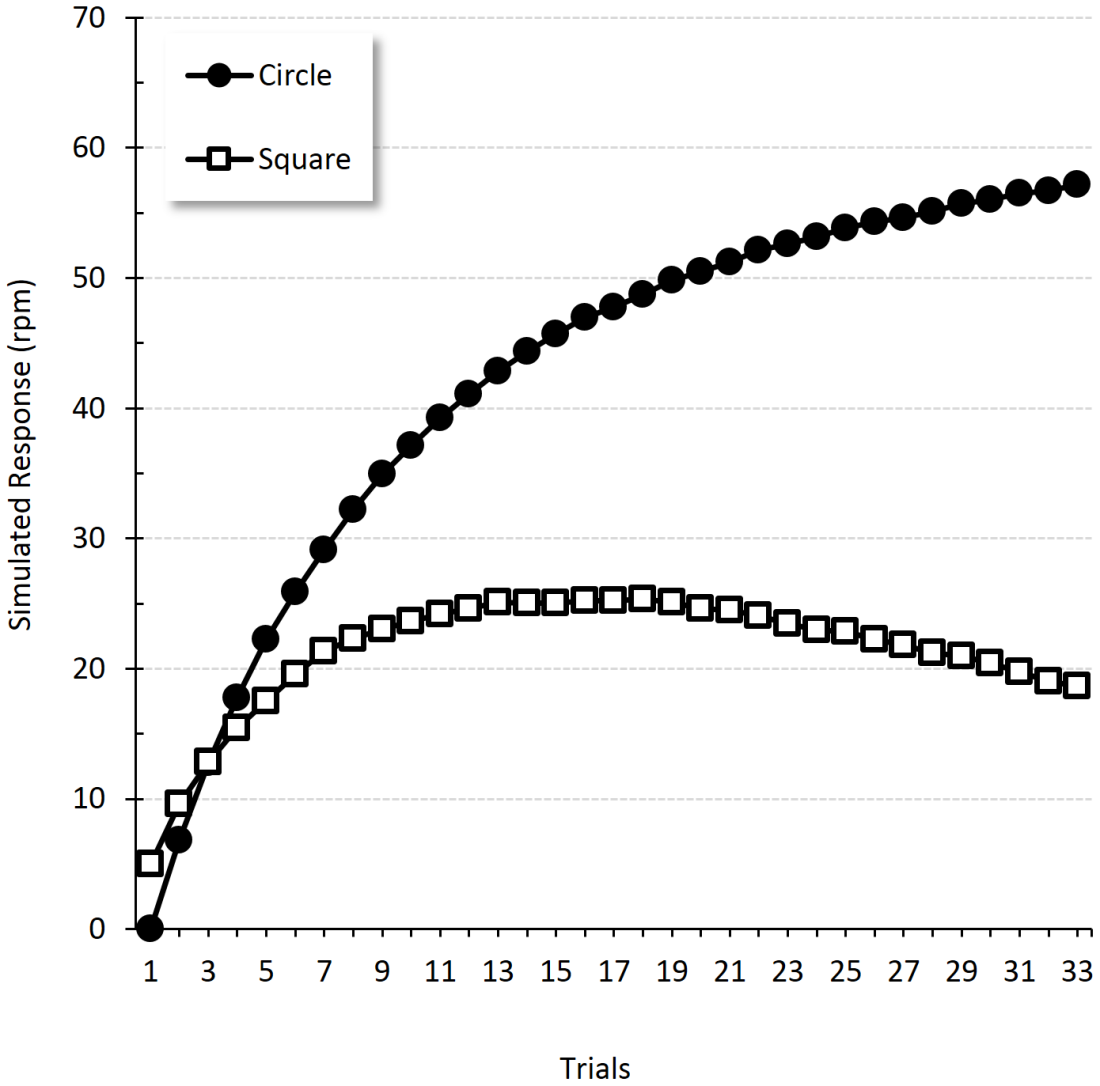
Stimulus generalization. Simulation of Pavlov's shape discrimination experiment showing responding to the positive (Circle) and negative (Square) stimuli across trials [36] (pp. 121).

Initially, both shapes engendered conditioned responding, showing that the animal generalized between the shapes. With enough training, however, the dog learned to salivate only when the circle was offered. Figure 6 displays the results of this simulation. As described by Pavlov, a small amount of responding is predicted for both stimuli at the beginning of the discrimination training. As the number of trials increases, responding is confined to the reinforced stimulus.

### Experiment 5. Renewal: Context effects

Renewal refers to a set of conditioning results that show a recovery of the conditioned response following extinction when the extinguished CS is tested in a context other than the one in which extinction occurred [65]–[66].

To test the Context-CS configuration algorithms in SSCC TD a classic appetitive renewal effect was simulated ([66], Experiment 1). Although there is plenty of evidence supporting the proposal that context summation cannot solely explain all instances of renewal [67]–[68], the contribution of the context associative strength to the phenomena is undeniable, particularly in those cases in which the test context is the same as the conditioning context.

Table 1 shows the design and parameters used for this simulation. Bouton and Peck's experiment consisted of three phases, and employed three groups. Phase 1 took place in Context ***A***; Groups ABA and AAA received 40 tone-food pairings whereas Group Control was exposed to 40 presentations of each of the stimuli, but unpaired. During Phase 2 all animals received 28 extinction trials with the tone, but where this training took place depended on the group. Group AAA received the extinction training in the conditioning context, Context ***A***; in Group ABA, the extinction occurred in a different context, Context ***B***; for half of the animals in Group Control, Phase 2 was given in Context ***A***, while for the remaining animals in this group Phase 2 took place in Context ***B***. During Phase 3, all animals received 24 tone test trials in Context ***A***. The physical identities of contexts ***A*** and ***B*** were counterbalanced across subjects.

The simulation we ran with parameters described in Table 1, using delay conditioning (ISI = 10 s) with a 10 s tone, and a variable ITI (792 s±402 s).

**Figure 7:**
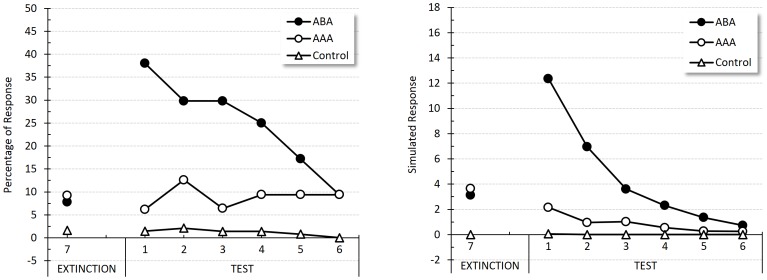
Renewal. Empirical (original measurement units) and simulated results during the last extinction block and during the test. Left panel reproduces an adaptation of Bouton and Peck's percentage of responses to the tone for Group ABA, Group AAA and Group Control in Experiment 1 [66]. Right panel shows the corresponding simulated responses per minute for each group and block of trials.

Figure 7 shows responding to the tone across blocks of 4 trials during the last extinction block and during the test in each group. The left panel displays the percentage of head-jerk response originally reported by Bouton and Peck, and the right panel the simulated responses for the context-tone compound. Visual inspection of both panels reveals that the simulated response rates match the pattern of the empirical results. There was a recovery of responding in Group ABA that received extinction in a different context from conditioning, and was tested in the original conditioning context. No such effect was observed either in Group AAA, for which both extinction and test occurred in the conditioning context, or in Group Control, which received the various phases in the same contexts as Group ABA, but experienced unpaired presentations of the two stimuli during the conditioning phase.

### Experiment 6. Conditional discriminations: Patterning vs. biconditional discriminations

An experiment published by Harris, Livesey, Gharaei and Westbrook [69] which investigated the learning rates of three conditional discriminations was simulated.

A conditional discrimination is a type of discrimination that cannot be solved based purely on the information provided by any given individual stimulus. In their experiment, Harris *et al*. compared the rates of learning a positive-patterning, a negative-patterning, and a biconditional discrimination. Positive patterning [13] involves the presentation of two stimuli (e.g., A and B) that are reinforced only when presented in compound (e.g., AB; i.e. AB+, A-, B-). Conversely, in a negative-patterning procedure two stimuli (e.g., C and D) are paired with a US when presented alone but when presented in compound (e.g., CD) the US is omitted (i.e., CD−, C+, D+) [70]. Animals trained on a positive-patterning discrimination learn to respond more to the compound AB than to each individual stimulus; in a negative-patterning procedure, C and D come to elicit a CR whereas the compound CD does not.

In a biconditional discrimination [13], [71] four stimuli are presented to form four different compounds AB, CD, AC and BD. AB and CD are paired with the US whereas AC and BD are not (i.e., AB+, CD+, AC−, BD−). Thus, each individual stimulus is equally associated with the reinforcer, so that the net associative strength of each compound, based on the summation principle, is also equivalent. However, animals do learn to respond more to the reinforced compounds than to the non-reinforced compounds.

While the summation assumption can easily explain the performance in positive patterning, it cannot predict differential responding in biconditional discriminations and in fact predicts the opposite results in negative-patterning procedures –more responding to CD than to C and D alone. As described above, in order to account for these types of non-linear discrimination, Wagner and Rescorla [14] proposed that when two stimuli are presented in compound, a configural cue may be formed.

Table 1 shows the design and parameters of the simulation of Harris *et al.*'s results. The experiment comprised two groups. Group Positive & Negative Patterning was trained on two concurrent patterning discriminations in which a compound AB was paired with food while its constituent stimuli A and B were not (Positive-Patterning discrimination) and the compound CD was consistently non-reinforced whereas its components C and D were paired with food (Negative-Patterning discrimination). Group Biconditional received the same number of stimuli, trials and reinforcement rate (50%) but was trained on a biconditional discrimination with the following stimulus arrangement: AB+, CD+, AC− and BD−. All stimuli were 30 s long, and presented in a delay conditioning procedure with an ISI of 30 s and an ITI of 120 s.

**Figure 8:**
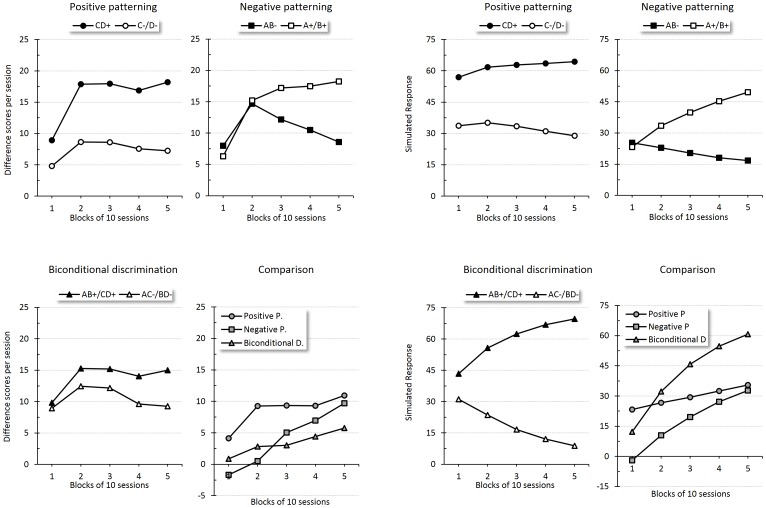
Conditional discriminations: patterning vs. biconditional discriminations. Empirical (original measurement units) and simulated results during discrimination training across sessions. Left panel shows an adaptation of Harris *et al.*'s CS-preCS difference scores for reinforced and non-reinforced trials in positive-patterning (top-left), negative-patterning (top-right) and biconditional discriminations (bottom-left) [69]. The bottom-right graph displays differential responding to positive and negative trials in each discrimination procedure. The right panel presents the corresponding simulated responses for each discrimination and comparison.

Harris *et al*.'s results are shown in the left panel of Figure 8. The right panel displays the corresponding simulated results. The top plots exhibit responding to the compounds and single stimuli for the positive-patterning (left) and negative-patterning (right) discriminations through training. Empirical and simulated results indicate a progressive increase in responding to the reinforced stimuli (or compounds) and a decrease in responding to the non-reinforced cues; this discriminative performance was acquired more quickly for the positive-patterning discrimination. The bottom left plot shows the acquisition of the biconditional discrimination and the bottom right plot displays a direct comparison of the discrimination rates across training, calculated as the difference in responding between positive and negative trials for each discrimination procedure. A quick inspection of these plots makes it obvious that there is a discrepancy between the empirical and simulated results. Contrary to the simulated results, which predict faster acquisition of the biconditional discrimination, in fact this was the hardest to learn. Harris *et al*. argued that a model that relies on configural cues to solve these discriminations (such as Wagner and Rescorla's and SSCC TD) would predict slower learning in negative patterning than in a biconditional discrimination, but that elemental models (e.g., [8]) depending on non-linear element-activation rules would predict that negative patterning is easier to learn –the result that Harris obtained. Consistent with this analysis, the simulation shows that a configural cue-based model can predict successful discrimination, but incorrectly predicts faster acquisition of the biconditional discrimination.

### Experiment 7. Summation: Assessing configural vs. elemental characteristics

Two main types of associative theories can be distinguished in terms of their approach to the problem of learning about compound stimuli. *Elemental* theories (e.g., [1]) envisage compound stimuli as cumulative sets of their constituent components whereas *configural* theories (e.g., [9]–[10]) regard compounds as distinctive stimuli. Compound associative strength is thus assumed to be the result of some sort of summation rule of the individual components' values in the former case, whereas in the latter the compounds' strength is derived from a given component/compound relationship, such as an explicit generalization rule. Elemental theories may also include unique configural cues that contribute to the compound's strength [14], to cope with instances of learning in which responding to the compound stimuli differs from what would be expected on the basis of simple component summation. Summation has been observed in many conditioning preparations (e.g., [72]), but experiments have been reported (e.g., [73]–[74]) in which summation does not seem to be found.

The SSCC TD model incorporates unique configural cues, preserving the summation assumption of an elemental approach to learning. To demonstrate this, we simulated an experiment precisely designed to assess summation in a situation in which only elemental theories are able to predict it ([51], Experiment 3).

Table 1 shows the design and simulation parameters used. Animals received conditioning trials to two 30 s compound stimuli AD and BC reinforced with food and non-reinforced presentations of a third compound AB (i.e., AD+, BC+, AB−) for the twenty two days of Phase 1. Phase 2 consisted of a single day of training identical to those of the previous phase except for the addition of 4 non-reinforced CD trials. During Phase 3, two days of re-training were given followed by a final test day in which 2 CD trials, and 1 non-reinforced trial with each of C and D were introduced during the second half of Phase 4 (otherwise identical to Phase 3).

Elemental theories anticipate that C and D will acquire considerably more associative strength than A and B; thus the joint presentation of C and D should result in summation, producing higher responding to CD than to AD or BC. In contrast, according to Pearce's model the compound of C and D should not produce summation, but rather less responding (or equal if an important role of the background or context stimuli is assumed) than that observed to AD and BC – because responding to CD will depend on the degree of generalization that CD receives from AD and BC, which in turn is based on the relative number of shared elements (just a fraction of each of the compounds).

We used the same design and temporal parameters, with a variable ITI (mean 120 s) as described above.

**Figure 9:**
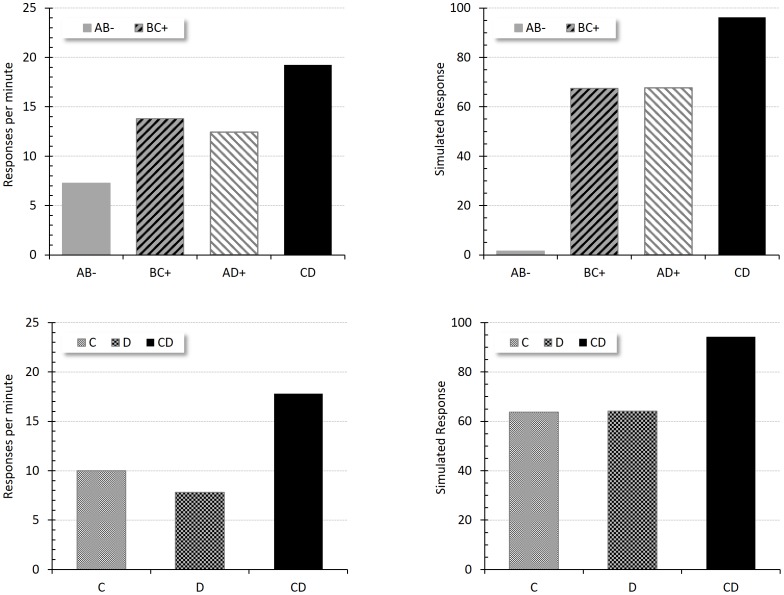
Summation. Empirical (original measurement units) and simulated results during the two tests of Rescorla's Experiment 3 [51]. The top-left panel is an adaptation of the data from the first test and shows responding during the trained compounds, AB-, BC+, and AD+ and during the CD test trials. The bottom-left panel displays the response during the final test to the individual stimuli C and D, as well as to the compound CD. The right panel presents the corresponding simulated responses for each test.

Figure 9 displays the test results of this experiment. The left panel shows the empirical results and the right panel the simulated response. In the top panel the simulated results correctly predict more responding to the compound CD than to AD or to BC, clearly reproducing the empirical findings of the Phase 2 test. On the bottom, a direct summation test shows more responding to CD than to either of its constituent stimuli, C and D, replicating accurately the empirical results in Phase 4.

### Experiment 8. Negative Occasion Setting

During the 80 s (but see [75] for early related studies), research into classical conditioning suggested that a stimulus could acquire other than simple elicited excitatory or inhibitory properties. More specifically, a stimulus can sometimes modulate (e.g., [76]) or facilitate (e.g., [77]) responding to another CS; in other words it can set the occasion for a response to occur (or not) to a particular stimulus. Occasion setters show properties that distinguish them from simple CSs and are sensitive to the temporal characteristics of the compound stimuli. Thus, for instance, in a feature-negative discrimination paradigm, in which a target stimulus is reinforced when presented alone but not reinforced when presented in compound with a second stimulus, animals learn to suppress (or inhibit) their response to the target when the second, feature stimulus is present. This procedure often endows the feature with different associative properties depending on the temporal mapping of the stimuli involved: when the stimuli in the compound are presented simultaneously, the feature acquires inhibitory properties as revealed by standard summation and retardation tests; when they are presented successively, however, the feature does not seem to acquire standard inhibitory properties; instead it gates the inhibitory properties of the target [78]–[79].

Holland [79] examined the effects that pairing simultaneous and serial features with the trained US would have on a feature-negative discrimination. If, following simultaneous feature-negative training, a feature controls responding to a target by acquiring an inhibitory association with the US, then the rate of acquisition of an excitatory association between the feature and the US should be reduced, compared to the rate at which conditioning to a non-inhibitory feature would develop [80]. Moreover, such excitatory conditioning should eliminate the feature's ability to inhibit responding to the trained target in a subsequent summation test. In contrast, if as a result of a serial feature-negative training the feature controls behavior by modulating a specific target-US association, the rate of acquisition of an excitatory association between the feature and the same US would not be retarded, and excitation training to the feature would not interfere with the modulation ability of the feature in a summation test.

Table 1 shows the design of Holland's Experiment 1. The experiment used a conditioned suppression procedure and all phases were conducted on a lever press baseline. Phase 1 lasted two sessions, each consisting of two 60 s presentations of a white noise (N) followed by a mild foot-shock. During Phase 2, two groups of rats were trained either on a simultaneous or a serial feature-negative discrimination. Each session in these two groups consisted of two 60 s reinforced presentations of the N and six 60 s nonreinforced presentations of a compound (LN) formed by a light (L) and N, randomly interspersed with a variable ITI (mean 780 s). In Group Sim the stimuli of the compound were presented simultaneously. In Group Ser the stimuli in the compound were presented serially, L followed by N. To equate the level of suppression to the nonreinforced stimuli 6 sessions were given to Group Sim and 24 to Group Ser. The remaining groups, Group Sim-C and Group Ser-C, served as comparison groups for Group Sim and Group Ser respectively. They received simple discrimination training consisting of two 60 s reinforced presentations of N and six nonreinforced 60 s presentations of L. The number of sessions in these groups was equated to that given to their counterparts, 6 sessions in Group Sim-C and 24 in Group Ser-C. During Phase 3 a retardation test was conducted, comprising 4 sessions consisting of two L-shock pairings. In Phase 4 the ability of L to inhibit responding to N was tested in a single session consisting of four nonreinforced N presentations and four nonreinforced presentations of the compound LN. The order of the trials was N, LN, LN, N, LN, N, N, LN. In Group Sim and Group Sim-C the stimuli of the compound LN were simultaneously presented whereas in Group Ser and Group Ser-C they were presented serially, L followed by N.

**Figure 10:**
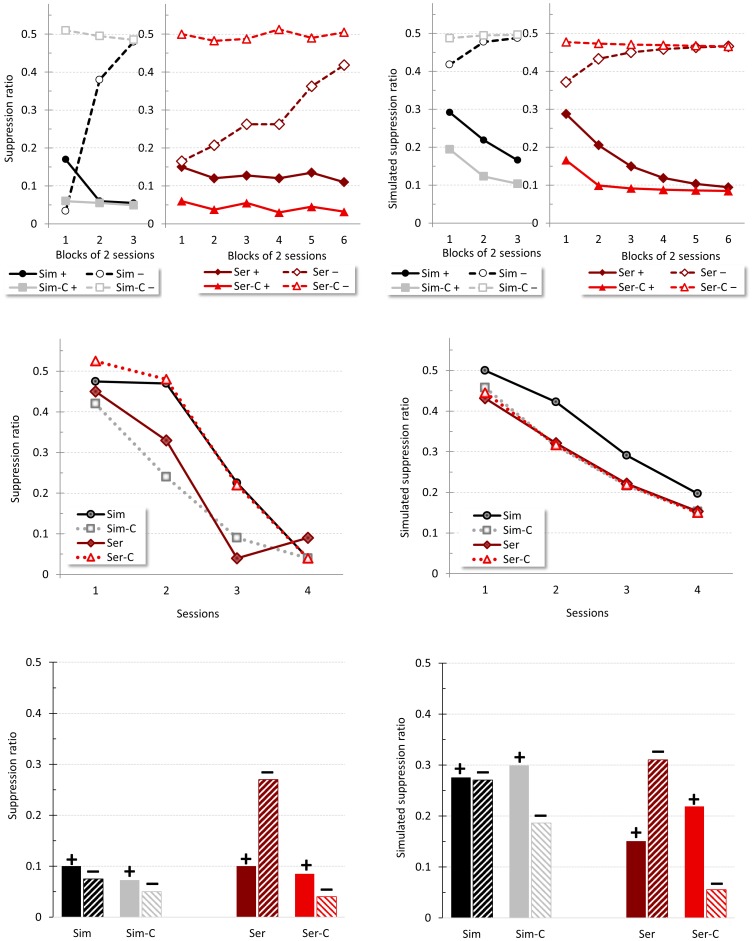
Negative occasion setting. Empirical (original measurement units, left panels) and simulated results (right panels) of Holland's Experiment 1 on feature-negative discrimination [79]. Top panels show discrimination training across blocks of 2 sessions in Group Sim (left), for simultaneous feature-negative training, and in Group Ser (right), for serial feature-negative training; and for their respective simple discrimination control groups (Group Sim-C and Group Ser-C). The symbol + represents positive trials (noise, N, trials) and the symbol – negative trials (compound light-noise, LN, in Group Sim and Group Ser, and L trials in Group Sim-C and Group Ser-C). Acquisition of responding to the light over four retardation test sessions in each group is represented in the middle panels. Bottom panels display results during the compound LN summation test following excitatory training to L.

Figure 10 shows Holland's results and their simulation. A simulated suppression ratio (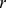) that parallels the empirical measure was computed by using the “maximum responses per minute” set in the simulation (100 rpm) instead of the baseline responding data. Thus, 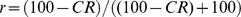. Computing a suppression ratio in this manner results in an equivalent behavioral pattern but lower general levels of suppression. Values closer to 0.5 (low levels of baseline response suppression) indicate poor conditioning– low CR – whereas values closer to 0 (high levels of baseline response suppression) represent strong conditioning – high CR. The empirical data from the discrimination results in Phase 2 are depicted in the top-left panel of Figure 10; the right panel displays the corresponding simulated results. The leftmost panel of the figures shows the data for the simultaneous groups and the rightmost segment the data for the serial groups. Consistent with the empirical findings, the simulation shows a strong suppression of responding across training during the positive trials (+) in which the noise was presented alone (Sim +, Sim-C +, Ser +, Ser-C +), whereas as training progressed suppression was gradually reduced during the negative (−) trials in which the compound light-noise was presented (Sim −, Sim-C −, Ser −, Ser-C −). Also consistent with the empirical results, discrimination in the control groups (Group Sim-C and Group Ser-C) appears to develop faster than in their respective counterpart experimental groups (Group Sim and Group Ser).

The results of Phase 3 are displayed in the middle panels of Figure 10, empirical on the left panel and simulated on the right panel. Critical to the hypothesis, conditioning to the light was retarded when the light was the simultaneously pre-trained feature (Group Sim) in comparison to the rate of conditioning in the control group (Group Sim-C). Retardation was not observed when the light was the serially trained feature (Group Ser vs. Group Ser-C). Notice that the empirical results did in fact show a reverse pattern, a facilitation of learning in Group Ser, an effect that was not planned or expected in Holland's experiment and was not replicated by the simulation.

The bottom left panel of Figure 10 depicts the empirical results during Phase 4 and the bottom right the corresponding simulation. In both cases, excitatory training of the negative feature only disrupted its modulation ability in a summation test in Group Sim, in which simultaneous feature-negative training was given. In this group, compound presentations of LN during the negative (−) trials no longer reduced suppression of responding when compared to the levels of suppression on the noise alone trials (+ trials). In contrast, the feature's ability to modulate behavior remained intact for the serially trained feature (Group Ser), considerably reducing suppression on − trials in relation to the observed levels on + trials. Remarkably, simulated results also seemed to enhance the difference observed between negative and positive trials in the control groups (Group Sim-C and Group Ser-C). Pairing two excitatory stimuli with the same US may result in an overexpectation effect that can be more evident in the control groups for which, unlike in the experimental groups, the stimuli had not been presented in compound before. Moreover, using the maximum response rate instead of the animals' baseline responding to compute suppression ratios results, as indicated above, in lower general levels of suppression, giving more room to uncover differences that could have been hidden due to a floor effect in the real conditions.

This simulation shows that SSCC TD predicts the correct pattern of response when comparing simultaneous vs. serial negative occasion-setting procedures. This pattern of response cannot, in principle, be explained by standard associative learning theories such as TD.

### Experiment 9. Serial structural discriminations

**Figure 11:**
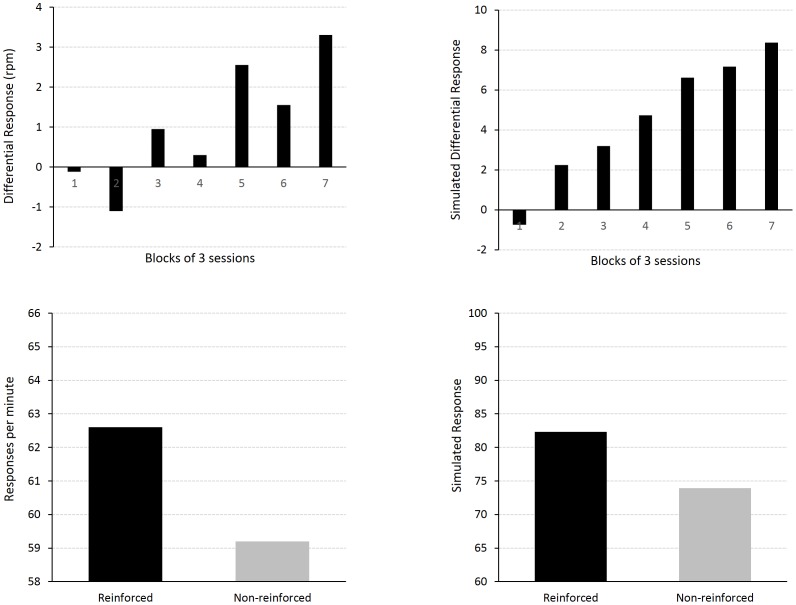
Serial structural discrimination. Empirical (original measurement units) and simulated results during Murphy *et al.*'s serial structural discrimination training (Experiment 1a) [46]. The top-left panel shows mean differential responses to the reinforced and non-reinforced trials across blocks of 3 sessions. In the bottom-left panel, mean responses per minute to the reinforced and to the non-reinforced compounds during the last training session are displayed. The right panel presents the corresponding simulated responses.

As a final test, we simulated a serial structural discrimination. In this type of procedure reinforced and non-reinforced configurations share the same set of elements, so that discrimination can only be attained on the basis of how the constituent elements are ordered. Murphy *et al.* [46] presented a serial structural design that fully equated the levels of associative strength of each element and of each compound: that is, the net associative strength of the compounds was the same regardless of the order of the stimuli involved. Table 1 shows the design and parameters used in the simulation of their Experiment 1a. Two 10 s stimuli were presented serially with a gap of 1 s between them; 4 different stimuli, (A, B, C, and D) and 8 different stimulus configurations (A→B, B→C, C→D, D→A, B→A, C→B, D→C, and A→D) were used and each was formed by an auditory cue and a visual cue. The stimuli were arranged such that each stimulus appeared in the first and the second positions an equal number of times and with the same probability of reinforcement; A→B, B→C, C→D, D→A were followed by reinforcement whereas B→A, C→B, D→C, and A→D were not. Each session consisted of 80 trials, 10 of each configuration, randomly distributed, with a variable ITI of 84 s. A total of 21 sessions was conducted. Discrimination was assessed by measuring the difference in responding on reinforced and non-reinforced trials during the second stimulus of the sequence. Murphy *et al.*'s results are shown in the left panel of Figure 11. The top panel depicts differential responding between the reinforced and non-reinforced trials expressed as responses per minute across 7 blocks of 3 sessions. The bottom panel displays mean conditioned responses during the reinforced and non-reinforced trials at the end of training. Analogous simulated results are presented in the right panel of the figure.

The pattern of simulated results is consistent with the empirical data. Discrimination between reinforced and non-reinforced stimulus sequences emerged with training. As with the experimental data, the discrimination is weak and reliable only after prolonged training. To our knowledge, performance in structural discriminations cannot be explained with standard associative frameworks, nor has it been tackled by configural approaches. This is thus the first model that has been able to account for this type of discrimination, which has been suggested to be at the core of higher cognitive phenomena such as rule learning (e.g., [81]).

## Discussion

Studies of classical conditioning have shown that animals are able to master discrimination problems which cannot be solved solely through learning about elemental stimuli. Performance on these tasks is controlled by configurations of stimuli rather than by simple stimulus-outcome associations. Understanding how stimulus configurations are represented and learned about is at the core of theoretical discussions in learning theory, and trial-based associative theories differ in how they approach this issue. Wagner and Rescorla [14], for example, proposed that a stimulus compound could be conceptualized as composed of the stimulus constituent elements plus an additional configural cue, whereas Pearce [9]–[10], [82] suggested that a stimulus activates a single configural node that represents the entire pattern of stimulation and that this node, not the primitive stimulus representation, becomes associated with the US. However, these theories all lack the advantage of real-time approaches and are thus only applicable to phenomena in which time and order are irrelevant. As the study of time-dependent discrimination procedures has flourished during recent decades, the inability of associative theories to cope with these types of tasks has become increasingly apparent. Integrating time within the associative framework is thus of urgent importance. For example, the results of timing studies such as Jennings *et al.* [64] are simply outside the scope of a standard trial-based associative analysis. The SSCC TD model is able to simulate them both at a trial level and in a real-time presentation of data.

Conversely, TD and its multiple instantiations have failed to incorporate means of representing stimulus configurations within their conceptualization, rendering them unable to explain performance on tasks that depend on compound rather than simple stimuli, as is the case in most learning paradigms. Consequently, TD models are ill-equipped to incorporate discrimination and generalization within their analysis. This drawback seriously limits the scope of TD. A representation of stimulus configurations enables SSCC TD to model largely known phenomena unaccounted for by previous TD models. For instance, stimulus generalization builds upon the presence of common elements between stimuli, which are assumed to be composed by common and distinctive elements functioning as a compound. A configuration representation that incorporates common elements between stimuli also allows for a more accurate reproduction of empirical data.

Additionally, as a real-time model, SSCC TD also offers a configural solution for *serial* feature discriminations such as those employed in the occasion setting study simulated in our results [80]. Configural interpretations of feature discriminations have been proposed before for *simultaneous* feature discriminations and for procedures in which the stimuli overlap at some point [83]–[84].

Moreover, since SSCC TD's representation of stimulus compounds incorporates Wagner and Rescorla's [14] notion of an added configural cue, the model is also able to deal with complex non-linear discriminations such as patterning discriminations. However, structural discriminations such as Murphy's *et al.* [46], in which discriminative performance can only be achieved by encoding the specific order in which the stimuli are presented in each configuration, require both a configural cue representation and a real-time framework. Thus these tasks are insoluble using trial-based or simple summation associative mechanisms. Alternative associative real-time models have been proposed that could provide a solution to the representation of stimulus compounds. For instance, in Brandon and Wagner's [11], [85]–[87] replacement elements theory, the representation of a compound of stimuli is assumed to involve both the addition and the subtraction (inhibition) of configural elements; error correction is estimated in a real time adaptation of the Rescorla and Wagner's algorithm by the discrepancy between the *memory traces* of the current outcome and the predicted outcome. That is, following the stimuli's offset, stimulus traces would determine real-time asymptotic distributions of associative strength that in turn would be used to compute each stimulus' associative strength. However, to our knowledge, these real time distributions of associative strength do not bear correspondence to a real-time output of the temporally distributed values of a stimulus (time-steps values), rendering the replacement elements model unable to simulate timing behavior (but see [88]). Schmajuk and collaborators' model ([89]–[93]) proposes a rather complicated neural architecture with multiple hidden units that could also account for some of these results. Harris' model [8] introduces a purely elemental approach to the representation of stimuli focused on the stimulus' elements or *microfeatures*. The stimulus *microfeatures* interact and their activation levels are modified according to a set of rules controlled by a limited capacity attentional buffer. In contrast, the SSCC TD model offers a simple, parsimonious way to represent configurations of stimuli in real-time and broadens significantly the range of phenomena accounted for in TD, allowing us to make insightful and accurate predictions for complex discriminations.

The results presented in this paper include examples of procedures anticipated by TD such as second-order conditioning, and blocking to illustrate the ability of SSCC TD to work with configurations and successfully predict performance in these tasks. Furthermore, the configural frame described here permits inclusion of contextual features within the experimental setting and to model context-dependent paradigms such as certain instances of the renewal effect [66].

Despite representing stimuli as configurations SSCC TD retains its elemental framework, which rests on the summation assumption of associative learning and on the notion of cue competition. Accurate simulation of Rescorla's [51] summation assessment backs this claim. Nonetheless, the incorporation of unique configural cues to the stimulus configuration representation goes beyond the strict linearity of the summation rule, enabling the model to predict performance in complex patterning discriminations. It must be noticed however that the degree of difficulty that each of these tasks poses was not fully satisfactorily predicted.

To summarize, the main contribution of the SSCC TD model is that it can accommodate many empirical results that depend on stimulus configurations and it does so using well-established, parsimonious concepts of classical conditioning theory, and in a real-time architecture. Being this a theoretical paper no new predictions, which will require empirical support, have been proposed. Nevertheless, a general prediction could be advanced which originates on the core associative principles underlying the model and its real-time structure: timing behavior and associative learning are entwined, and therefore, basic associative effects such as those derived from cue competition and the summation assumption, will also have an impact on timing. As an example, the SSCC TD model predicts that acquisition of a timing response to a stimulus will develop faster and with an initial steeper slope when the stimulus is presented paired with and inhibitor than when paired with a neutral CS. On the other hand, temporal factors may restrict the scope of associative phenomena. For instance, blocking of a stimulus presented serially prior the blocking stimulus may be largely reduced – or even reversed – due to the development of second-order associations resulting from the temporal characteristics of the TD algorithm (see [94] for an early suggestion of this effect).

The work presented in this paper may be of relevance for different lines of research. First, although the SSCC TD model has focused on behavioral data, it is well known that TD has been proposed as a computational model of classical conditioning at both behavioral and neural levels. The neuroscience community may benefit from using an extended representation of TD that can throw some light into the analysis of neural data. Second, as a reinforcement learning algorithm, TD can accommodate results from both classical and operant conditioning. In its current form SSCC TD is restricted to classical conditioning phenomena but in principle it could be applied to the study of instrumental learning and decision-making. Third, our model is contextualized within associative theories of learning and compared against trial-based and real-time associative models –which it enhances. The results presented in the paper may trigger a healthy debate about the relative strengths and weaknesses of alternative approaches to learning theory such as those embodied in Bayesian and information models.

## Supporting Information

Appendix S1 **Example with step by step computations of the SSCC TD model and a glossary of symbols and parameters.**
(DOCX)

Design Datasets S1 **Files containing the design and parameters for each experiment to be opened with the SSCC TD Simulator.**
(ZIP)

Simulator Quick Guide S1 **A nutshell guide to use the SSCC TD Simulator.**
(DOCX)

## References

[1] Rescorla RA, Wagner AR (1972) A theory of Pavlovian conditioning: The effectiveness of reinforcement and non-reinforcement. In Black AH, Prokasy WF, editors, Classical Conditioning II: Current Research and Theory. New York: Appleton-Century-Crofts, pp. 64–99.

[2] Sutton RS, Barto AG (1987) A temporal-difference model of classical conditioning. In Proceedings of the Ninth Annual Conference of the Cognitive Science Society, pp. 355–378.

[3] Sutton RS, Barto AG (1990) Time-derivative models of Pavlovian reinforcement. In Gabriel M, Moore JW, editors, Learning and computational neuroscience. Cambridge, MA: MIT Press, pp. 497–537.

[4] Alonso E, Mondragón E, Fernández A (2012) A Java simulator of Rescorla and Wagner's prediction error model and configural cue extensions. Computer Methods and Programs in Biomedicine 108: 346–355. 22420931 10.1016/j.cmpb.2012.02.004

[5] Kehoe EJ, Schreurs BG, Graham P (1987) Temporal primacy overrides prior training in serial compound conditioning of the rabbit's nictitating membrane response. Animal Learning Behavior 15: 455–464.

[6] Barto AG, Sutton RS (1982) Simulation of anticipatory responses in classical conditioning by a neuron-like adaptive element. Behavioral Brain Research 4: 221–235. 10.1016/0166-4328(82)90001-8 6277346

[7] Bouton ME, Doyle-Burr C, Vurbic D (2012) Asymmetrical Generalization of Conditioning and Extinction From Compound to Element and Element to Compound. Journal of Experimental Psychology: Animal Behavior Processes 38: 381–393. 22924827 10.1037/a0029726 PMC4026942

[8] Harris JA (2006) Elemental representations of stimuli in associative learning. Psychological Review 113: 584–605. 16802882 10.1037/0033-295X.113.3.584

[9] Pearce JM (1987) A model for stimulus generalization in Pavlovian conditioning. Psychological Review 94: 61–73. 3823305

[10] Pearce JM (1994) Similarity and discrimination: A selective review and a connectionist model. Psychological Review 101: 587–607. 7984708 10.1037/0033-295x.101.4.587

[11] Wagner AR, Brandon SE (2001) A componential theory of Pavlovian conditioning. In Mowrer RR, Klein SB, editors, Handbook of contemporary learning theories. Mahwah, NJ: Erlbaum, pp. 23–64.

[12] Ludvig EA, Sutton RS, Kehoe EJ (2012) Evaluating the TD model of classical conditioning. In Special Issue on Computational Models of Classical Conditioning, Learning Behavior Alonso E, Schmajuk N, editors, 40: 305–319. 10.3758/s13420-012-0082-6 22927003

[13] Rescorla RA (1972) “Configural” conditioning in discrete-trial bar pressing. Journal of Comparative and Physiological Psychology 79: 307–317. 5025999 10.1037/h0032553

[14] Wagner AR, Rescorla RA (1972) Inhibition in Pavlovian conditioning: Application of a theory. In Boakes RA, Halliday MS, editors, Inhibition and Learning. New York: Academic Press, pp. 301–336.

[15] Moore J, Choi J, Brunzell D (1998) Predictive timing under temporal uncertainty: the TD model of the conditioned response. In Rosenbaum D, Collyer A, editors, Timing of Behavior: Neural, Computational, and Psychological Perspectives. Cambridge, MA: MIT Press, pp. 3–34.

[16] Baker TW (1968) Properties of Compound Conditioned Stimuli and their Components. Psychological Bulletin 70: 611–625.

[17] Razran G (1965) Empirical codifications and specific theoretical implications of compound-stimulus conditioning: Perception. In Prokasy WF editor, Classical conditioning. New York: Appleton-Century-Crofts, pp. 226–248.

[18] Wickens DD (1959) Conditioning to complex stimuli. American Psychologist 14: 180–188.

[19] Ludvig EA, Bellemare MG, Pearson KG (2011) A primer on reinforcement learning in the brain: Psychological, computational, and neural perspectives. In Alonso E, Mondragón E, editors, Computational Neuroscience for Advancing Artificial Intelligence: Models, Methods and Applications. Hershey, PA: IGI Global, pp. 111–144.

[20] Montague PR, Dayan P, Sejnowski TJ (1996) A framework for mesencephalic dopamine systems based on predictive Hebbian learning. The Journal of Neuroscience 16: 1936–1947. 8774460 10.1523/JNEUROSCI.16-05-01936.1996 PMC6578666

[21] Niv Y (2009) Reinforcement learning in the brain. Journal of Mathematical Psychology 53: 139–154.

[22] Schultz W (2006) Behavioral theories and the neurophysiology of reward. Annual Review of Psychology 57: 87–115. 10.1146/annurev.psych.56.091103.070229 16318590

[23] Schultz W, Dayan P, Montague PR (1997) A neural substrate of prediction and reward. Science 275: 1593–1599. 9054347 10.1126/science.275.5306.1593

[24] Schultz W (2010) Dopamine signals for reward value and risk: basic and recent data. Behavioral and Brain Functions 6: 6–24. 20416052 10.1186/1744-9081-6-24 PMC2876988

[25] Schultz W, Dickinson A (2000) Neuronal Coding of Prediction Errors. Annual Review of Neuroscience 23: 473–500. 10.1146/annurev.neuro.23.1.473 10845072

[26] Amundson J, Miller RR (2008) CS–US temporal relations in blocking. Learning Behavior 36: 92–103. 18543710 10.3758/lb.36.2.92 PMC2587234

[27] Church RM, Kirkpatrick K (2001) Theories of conditioning and timing. In Mowrer RR, Klein SB, editors, Contemporary learning: Theory and Applications. Hillsdale, NJ: Erlbaum, p. 211–253.

[28] Jennings DJ, Kirkpatrick K (2006) Interval duration effects on blocking in appetitive conditioning. Behavioural Processes 71: 318–329. 16378697 10.1016/j.beproc.2005.11.007

[29] Cohen JD, Servan-Schreiber D (1992) Context, cortex, and dopamine: a connectionist approach to behavior and biology in schizophrenia, Psychological Review. 99: 45–77. 10.1037/0033-295x.99.1.45 1546118

[30] Montague PR, Hyman SE, Cohen JD (2004) Computational roles for dopamine in behavioural control. Nature 431 (7010): 760–767. 15483596 10.1038/nature03015

[31] Smith A, Li M, Becker S, Kapur S (2006) Dopamine, prediction error, and associative learning: a model-based account. Network: Computation in Neural Systems 17: 61–84. 10.1080/09548980500361624 16613795

[32] Estes WK (1950) Toward a statistical theory of learning. Psychological Review 57: 94–107.

[33] Hull CL (1943) Principles of behavior. New York: Appleton-Century-Crofts.

[34] Thorndike EL (1911) Animal intelligence: Experimental studies. New York: Macmillan.

[35] Wagner AR (1981) SOP: A model of automatic memory processing in animal behavior. In N. ESpear R. RMiller, editors, Information processing in animals: Memory mechanism. Hillsdale, NJ: Erlbaum, pp. 95–128.

[36] Pavlov IP (1927) Conditioned reflexes. Oxford: Oxford University Press.

[37] Rescorla RA (1976) Stimulus generalization: Some predictions from a model of Pavlovian conditioning. Journal of Experimental Psychology: Animal Behavior Processes 2: 88–96. 1249526 10.1037//0097-7403.2.1.88

[38] Razran G (1939) Studies in Configural Conditioning: I. Historical and Preliminary Experimentation. The Journal of General Psychology 21: 307–330.

[39] Rescorla RA (1973) Evidence for “unique stimulus” account of configural conditioning. Journal of Comparative and Physiological Psychology 85: 331–338.

[40] Wagner AR (1971) Elementary Associations. In Kendler HH, Spence JT, editors, Essays in Neobehaviorism: A Memorial Volume to Kenneth W. Spence. New York: Appleton-Century-Crofts, pp. 187–213.

[41] Whitlow JW Jr, Wagner AR (1972) Negative patterning in classical conditioning: Summation of response tendencies to isolable and configural components. Psychonomic Science 27: 299–301.

[42] Brandon SE, Vogel EH, Wagner AR (2000) A componential view of configural cues in generalization and discrimination in Pavlovian conditioning. Behavioural Brain Research 110: 67–72. 10802304 10.1016/s0166-4328(99)00185-0

[43] McNally GP, Pigg M, Weidemann G (2004) Blocking, Unblocking, and Overexpectation of Fear: A Role for Opioid Receptors in the Regulation of Pavlovian Association Formation. Behavioral Neuroscience 118: 111–120. 14979787 10.1037/0735-7044.118.1.111

[44] Woods AM, Bouton ME (2006) D-cycloserine facilitates extinction but does not eliminate renewal of the conditioned emotional response. Behavioral Neuroscience 120: 1159–1162. 17014266 10.1037/0735-7044.120.5.1159

[45] Aggleton JP, Amin E, Jenkins TA, Pearce JM, Ward-Robinson J (2011) Lesions in the anterior thalamic nuclei of rats do not disrupt acquisition of stimulus sequence learning. The Quarterly Journal of Experimental Psychology 64: 65–73. 20680891 10.1080/17470218.2010.495407 PMC4255482

[46] Murphy RA, Mondragón E, Murphy VA, Fouquet N (2004) Serial order of conditional stimuli as a discriminative cue for Pavlovian conditioning. Behavioural Processes 67: 303–311. 15499680 10.1016/j.beproc.2004.05.003

[47] Weisman RG, Wasserman EA, Dodd PWD, Larew MB (1980) Representation and retention of two-event sequences in pigeons. Journal of Experimental Psychology: Animal Behavioral Processes 6: 312–325.

[48] Kehoe EJ, Gormezano I (1980) Configuration and combination laws in conditioning with compound stimuli. Psychological Bulletin 87: 351–378. 7375603

[49] Holland PC (1983) Occasion-setting in Pavlovian feature positive discriminations. In Commonds ML, Herrnstein RJ, Wagner AR, editors, Quantitative Analyses of Behavior: Discrimination Processes, Vol. 4 . Ballinger, New York, pp. 183–206.

[50] Sutherland RJ, Rudy JW (1989) Configural association theory: The role of the hippocampal formation in learning, memory, and amnesia. Psychobiology 17: 129–144.

[51] Rescorla RA (1997) Summation: Assessment of a configural theory. Animal Learning Behavior 25: 200–209.

[52] Fraisse P (1984) Perception and Estimation of Time. Annual Review of Psychology Vol. 35: 1–37. 10.1146/annurev.ps.35.020184.000245 6367623

[53] Gershman SJ, Blei D, Niv Y (2010) Context, learning and extinction. Psychological Review 117: 197–209. 20063968 10.1037/a0017808

[54] Haselgrove M, Robinson J, Nelson A, Pearce JM (2008) Analysis of an ambiguous-feature discrimination. The Quarterly Journal of Experimental Psychology 61: 1710–1725. 18942036 10.1080/17470210701680746

[55] Pearce JM, George D, Redhead ES (1998) The Role of Attention in the Solution of Conditional Discriminations. In Schmajuk NA, Holland PC, editors, Occasion Setting: Associative learning and cognition in animals. Washington DC: American Psychological Association, pp. 249–275.

[56] Schmajuk N, Alonso E (2012) Special Issue on Computational Models of Classical Conditioning. Learning & Behavior 40.. 10.3758/s13420-012-0081-7 22926998

[57] Holland PC, Rescorla RA (1975) Second-order conditioning with food unconditioned stimulus. Journal of Comparative and Physiological Psychology 88: 459–467. 1120816 10.1037/h0076219

[58] Rizley RC, Rescorla RA (1972) Associations in second-order conditioning and sensory preconditioning. Journal of Comparative and Physiological Psychology 81: 1–11. 4672573 10.1037/h0033333

[59] Tabone CJ, de Belle JS (2011) Second-order conditioning in Drosophila. Learning Memory 18: 250–253. 21441302 10.1101/lm.2035411 PMC3072777

[60] Allen MT, Padilla Y, Gluck MA (2002) Blocking in rabbit eyeblink conditioning is not due to learned inattention: indirect support for an error correction mechanism of blocking. Integrative Physiological Behavioral Science 37: 254–264. 12645843 10.1007/BF02734248

[61] Mackintosh N (1973) Stimulus selection: Learning to ignore stimuli that predict no change in reinforcement. In Hinde R, Stevenson-Hinde J, editors, Constraints on Learning: Limitations and Predispositions. New York: Academic Press, pp. 75–96.

[62] Solomon PR (1980) A time and place for everything? temporal processing views of hippocampal function with special reference to attention. Physiological Psychology 8: 254–261.

[63] Gluck MA, Allen MT, Myers CE, Thompson RF (2001) Cerebellar substrates for error-correction in motor conditioning. Neurobiology of Learning and Memory 76: 314–341. 11726240 10.1006/nlme.2001.4031

[64] Jennings D, Alonso E, Mondragón E, Franssen M. Bonardi C (2013) The Effect of Stimulus Distribution Form on the Acquisition and Rate of Conditioned Responding: Implications for Theory. Journal of Experimental Psychology: Animal Behavior Processes 39: 233–248. 23627799 10.1037/a0032151

[65] Bouton ME, Bolles RC (1979) Contextual control of the extinction of conditioned fear. Learning and Motivation 10: 445–466.

[66] Bouton ME, Peck CA (1989) Context effects on conditioning, extinction, and reinstatement in an appetitive conditioning preparation. Animal Learning Behavior 7: 88–98.

[67] Bouton ME, King DA (1983) Contextual control of the extinction of conditioned fear: tests for the associative value of the context. Journal of Experimental Psychology: Animal Behavior Processes 9: 248–265. 6886630

[68] Bouton ME, Swartzentruber D (1986) Analysis of the associative and occasion-setting properties of contexts participating in a Pavlovian discrimination. Journal of Experimental Psychology: Animal Behavior Processes 12: 333–350.

[69] Harris JA, Livesey EJ, Gharaei S, Westbrook RF (2008) Negative Patterning Is Easier Than a Biconditional Discrimination. Journal of Experimental Psychology: Animal Behavior Processes 34: 494–500. 18954233 10.1037/0097-7403.34.4.494

[70] Rescorla RA, Grau JW, Durlach PJ (1985) Analysis of the unique cue in configural discriminations, Journal of Experimental Psychology: Animal Behavior Processes. 11: 356–366. 4009124

[71] Saavedra MA (1975) Pavlovian compound conditioning in the rabbit. Learning and Motivation 6: 314–326.

[72] Kehoe EJ (1986) Summation and configuration in conditioning of the rabbit's nictitating membrane response. Journal of Experimental Psychology: Animal Behavior Processes 12: 186–195. 7175444

[73] Aydin A, Pearce JM (1995) Summation in autoshaping with short- and long-duration stimuli. Quarterly Journal of Experimental Psychology 488: 215–234.

[74] Rescorla RA, Coldwell SE (1995) Summation in autoshaping. Animal Learning Behavior 23: 314–326.

[75] Skinner BF (1938) The behavior of organisms: An experimental analysis. New York: D. Appleton-Century Company, Inc.

[76] Holland PC (1985) The nature of conditioned inhibition in serial and simultaneous feature negative discrimination training. In R. RMiller N. ESpear, editors, Information processing in animals: Conditioned inhibition (pp. 267–298) Hillsdale, NJ: Lawrence Erlbaum Associates, Inc.

[77] Rescorla RA (1985) Conditioned inhibition and facilitation. In Miller RR, Spear NE, editors, Information processing in animals: Conditioned inhibition. Hillsdale, NJ: Lawrence Erlbaum Associates, pp. 299–326.

[78] Bouton ME, Nelson JB (1994) Context-specificity of target versus feature inhibition in a feature-negative discrimination. Journal of Experimental Psychology: Animal Behavior Process 20: 51–65. 8308493

[79] Holland PC (1984) Differential effects of reinforcement of an inhibitory feature after serial and simultaneous feature negative discrimination training. Journal of Experimental Psychology: Animal Behavior Process 10: 461–475. 6491607

[80] Rescorla RA (1969) Pavlovian conditioned inhibition. Psychological Bulletin 72: 77–94.

[81] Murphy RA, Mondragón E, Murphy VA (2008) Rule learning by rats. Science 319(5871): , 1849–1851. 10.1126/science.1151564 18369151

[82] Pearce JM (2002) Evaluation and development of a connectionist theory of configural learning. Animal Learning & Behavior 30: 73–95. 12141138 10.3758/bf03192911

[83] Pearce JM, George D, Redhead ES (1998) The Role of Attention in the Solution of Conditional Discriminations. In Schmajuk NA, Holland PC, editors, Occasion Setting: Associative learning and cognition in animals. Washington DC: American Psychological Association, pp. 249–275.

[84] Honey RC, Watt A (1999) Acquired relational equivalence between contexts and features Journal of Experimental Psychology: Animal Behavior Processes. 25: pp. 324–333. 10.1037//0097-7403.24.3.325 9679308

[85] Brandon SE, Wagner AR (1998) Occasion setting: Influences of conditioned emotional responses and configural cues. In Schmajuk NA, Holland PC, editors, Occasion Setting: Associative learning and cognition in animals. Washington DC: American Psychological Association, pp. 343–382.

[86] Wagner AR (2003) Context-sensitive elemental theory. Quarterly Journal of Experimental Psychology 56B: 7–29. 10.1080/02724990244000133 12623534

[87] Wagner AR, Brandon SE (2001) A componential theory of Pavlovian conditioning. In Mowrer RR, Klein SB, editors, Handbook of contemporary learning theories. Mahwah NJ: Lawrence Erlbaum Associates, pp. 23–64.

[88] Vogel EH, Brandon SE, Wagner AR (2003) Stimulus representation in SOP: II. An application to inhibition of delay. Behavioural Processes 62: 27–48. 12729967 10.1016/s0376-6357(03)00050-0

[89] Kutlu MG, Schmajuk NA (2012) Solving Pavlov's puzzle: attentional, associative, and flexible configural mechanisms in classical conditioning. Learning & Behavior 40: 269–91. 22927001 10.3758/s13420-012-0083-5

[90] Schmajuk NA, DiCarlo JJ (1992) Stimulus configuration, classical conditioning, and the hippocampus. Psychological Review 99: 268–305. 1594726 10.1037/0033-295x.99.2.268

[91] Schmajuk NA, Lam YW, Gray JA (1996) Latent inhibition: A neural network approach. Journal of Experimental Psychology: Animal Behavior Processes 22: 321–349. 8691162 10.1037//0097-7403.22.3.321

[92] Schmajuk NA, Larrauri JA (2006) Experimental challenges to theories of classical conditioning: application of an attentional model of storage and retrieval. Journal of Experimental Psychology: Animal Behavior Processes 32: 1–20. 16435961 10.1037/0097-7403.32.1.1

[93] Schmajuk NA, Lamoureux JA, Holland PC (1998) Occasion setting: a neural network approach. Psychological Review 105: 3–32. 9450370 10.1037/0033-295x.105.1.3

[94] Aguado L, López M, Lillo J (1989) Blocking with Serial Compound Stimuli: The Role of Local Context and Second-order Associations. Quarterly Journal of Experimental Psychology 41B: 3–19.

