# Supplementary material for: SSCC TD: A Serial and Simultaneous Configural-Cue Compound Stimuli Representation for Temporal Difference Learning
Source: PLoS One. 2014 Jul 23;9(7):e102469. doi: 10.1371/journal.pone.0102469 (PMC4108321; doi:10.1371/journal.pone.0102469)
Supplement: Appendix S1 — Example with step by step computations of the SSCC TD model and a glossary of symbols and parameters. (DOCX) [file pone.0102469.s001.docx]

**Appendix**

Let us assume two AB reinforced trials where A is 3s long and B 2s, and both stimuli offset at the time of reinforcement as depicted in Figure S1.


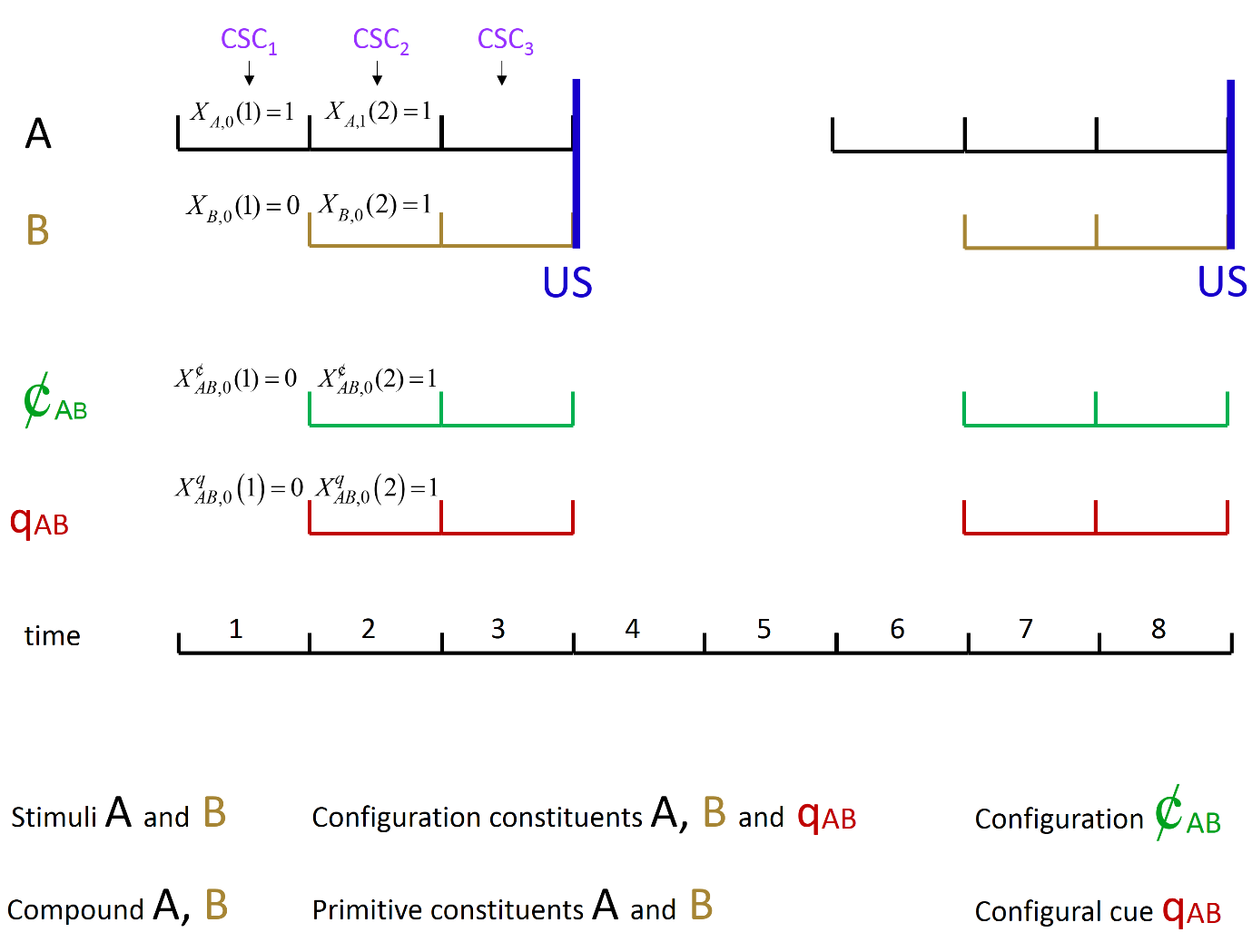


**Figure S1. Step by step calculation of the presence of the elements of a configuration.**

Schematics of the calculation of the presence of the components of the primitive constituents, configural cue and configurations during the first time-steps of an AB trial.

Trial 1

**1^st^ time unit**

***Stimulus presence***: A is present during the first time-step on trial 1 and B is not, thus $X_{A,0}(1)=1$ and$X_{B,0}(1)=0$. Notice that stimulus components use 0 based indexing, such that the first component is number 0.

***Configuration presence***: At each time-step, the presence of a configuration ¢AB (*m* = AB) is assessed in Equation 4:

$X_{m}^{\text{¢}}\left(t\right)=\prod_{a} \sum_{b} X_{a,b}\left(t\right)-\min\left(1, \sum_{i} \sum_{j} X_{i,j}\left(t\right)\right); if X_{m}^{\text{¢}}\left(t\right)\geq0, otherwise 0$.

Since there is no other stimuli present outside the target configuration at this time-step, $\sum_{i} \sum_{j} X_{i,j}\left(t\right)=0$.

Thus, $X_{AB}^{\text{¢}}(1)=\left(X_{A,0}\left(1\right)\right)\left(X_{B,0}\left(1\right)\right)-\min\left(1,\sum_{i} \sum_{j} X_{i,j}\right)=1\times0-min\left(1,0\right)=0$, that indicates that the configuration is not present at *t* = 1.

Since the configuration is not present, clearly none of the configuration components will be. Equation 5, $X_{m,0}^{\text{¢}}\left(t\right)=\max\left[0,X_{m}^{\text{¢}}\left(t\right)-X_{m}^{\text{¢}}\left(t-1\right)\right]$, is used to confirm this. Letting$X_{AB}^{\text{¢}}(1)=0$, results in $X_{AB,0}^{\text{¢}}\left(1\right)=\max\left[0,X_{AB}^{\text{¢}}\left(1\right)-X_{AB}^{\text{¢}}\left(0\right)\right]=\max\left[0,0-0\right]=0$.

Since by Equation 4,$X_{AB}^{\text{¢}}\left(1\right)=0$, Equation 6 $X_{m,n}^{\text{¢}}\left(t\right)=X_{m}^{\text{¢}}\left(t\right)\cdot X_{m,n-1}^{\text{¢}}\left(t-1\right)$, becomes$X_{AB,0}^{\text{¢}}\left(1\right)=X_{AB}^{\text{¢}}\left(1\right)\cdot X_{AB,0-1}^{\text{¢}}\left(1-1\right)$, and it is of necessity 0 for all *n*. As a result no configuration is assumed present at component *n* = 1.

Equations 5 and 6 are necessary because Equation 6 is framed as relying on the presence of the previous component on the prior time-step what implies that it is effectively recursive, and as such it requires a base case for the first component that is provided by Equation 5.

***Configural cue presence***: Since the configuration is not present, no configural cue is instantiated, thus$X_{AB,0}^{q}\left(1\right)=0$.

***Stimulus associative strength value***: by definition the predicted associative value for each stimuli at time-step 1 is zero. $V_{A,0}(1)=0$; $V_{B,0}(1)=0$ and .

***Configuration associative strength value****:* no configuration is present at this point, thus $V_{AB}^{\text{¢}}\left(t\right)=0$

**2^nd^ time unit**

***Stimulus presence***: Both A and B are present in time-step 2. Since the first CSC component of A appeared in time-step 1, now it is the second CSC component of A that is represented$X_{A,1}(2)=1$ ; for B however it is the first component that appears in time-step 2. So$X_{B,0}(2)=1$. The CSC representation assumes that there is a direct mapping between a time-step at the chosen level of granularity, and a component. While a stimulus continues, this implies that new components are added to the sequence that represents the stimulus. Similarly, components are active for only a single time-step, such that $X_{A,0}\left(2\right)=0$, i.e. the first component of A is now inactive.

***Configuration presence***: We use Equation 4 to calculate the presence of the configuration AB. Thus,$X_{AB}^{\text{¢}}\left(2\right)=\left(X_{A,0}\left(2\right)+X_{A,1}\left(2\right)\right)\left(X_{B,0}\left(2\right)+X_{B,1}\left(2\right)\right)-0=\left(0+1\right)\left(0+1\right)-0=1$. Since a value of 1 is obtained the configuration presence is established.

Following Equation 5 then gives the presence of the first component of the AB configuration as follows:

$X_{AB,0}^{\text{¢}}\left(2\right)=\max\left[0,X_{AB}^{\text{¢}}\left(2\right)-X_{AB}^{\text{¢}}\left(1\right)\right]=\max\left[0,1-0\right]=1$, that is, the configuration component of the ¢AB present in *t* = 2 is the initial component $X_{AB,0}^{\text{¢}}\left(2\right)=1$ .

Equation 6 is redundant at the moment of initialization, at which point *n* = 1.

***Configural cue presence****:* Since $X_{AB,0}^{\text{¢}}(2)=1$ by Equation 7, as in $If X_{m,n}^{\text{¢}}\left(t\right)=1 then X_{m,n}^{q}\left(t\right)=1$,

a unique configural cue, q_AB_, in time-step 2 is assumed to be formed and a presence value of 1 is assigned to it $X_{AB,0}^{q}\left(2\right)=1$ .

***Stimulus associative strength value***:

Let us assign some values to the constant parameters: $\gamma=0.97$;$\rho=0.97$;$\beta=0.5$; $\alpha_{A}=0.2;$ $\alpha_{B}=0.2;$ $\alpha_{q_{AB}}=0.04$.

Now we need first to update the learning rule following in Equation 1,

$$\delta V_{i,j}(t+1)=X_{0}(t+1)+ \gamma\left[\sum_{i} \sum_{j} X_{i,j}(t+1) V_{i,j}(t)\right]- \left[\sum_{i} \sum_{j} X_{i,j}(t) V_{i,j}(t)\right]$$

Since no reinforcer has yet been presented $X_{0}(2)=0$

A configural cue, q_AB_, was assumed above, and thus it has to be included in the calculations:

$$\delta V_{A,1}(2)=0+ 0.97\left[1\times0+1\times0+0\times0\right]- \left[1\times0+0\times0+0\times0\right]=0$$

$$\delta V_{B,0}(2)=0+ 0.97\left[0\times0+1\times0+0\times0\right]- \left[0\times0+1\times0+0\times0\right]=0$$

$$\delta V_{AB,0}^{q}(2)=0+ 0.97\left[0\times0+1\times0+0\times0\right]- \left[0\times0+1\times0+0\times0\right]=0$$

Equation 3, $e_{i,j}\left(t+1\right)=\text{min}\left[1, \rho\gamma e_{i,j}\left(t\right)+X_{i,j}\left(t\right)\right]$, is now used to calculate the eligibility trace *e* of each stimulus component at *t*. Notice that $e_{i,j}\left(0\right)=0$. Hence,

$$e_{A,1}\left(2\right)=\text{min}\left[1, 0.97\times0.97\times0+1\right]=1$$

$$e_{B,0}\left(2\right)=\text{min}\left[1, 0.97\times0.97\times0+0\right]=0$$

$$e_{AB,0}^{q}\left(2\right)=\text{min}\left[1, 0.97\times0.97\times0+0\right]=0$$

Finally we calculate the associative strength with Equation 2

$V_{i,j}(t+1)=V_{i,j}(t)+\beta\alpha_{i}\delta V_{i,j}(t+1)e_{i,j}(t+1)$. Being the values of $V_{i,j}(1)=0$ and $e_{i,j}(2)=0$,

$V_{A,1}(2)=0+0.5\times0.2\times0\times1=0$;

$V_{B,0}(2)=0+0.5\times0.2\times0\times0=0$; and

$$V_{AB,0}^{q}(2)=0+0.5\times0.04\times0\times0=0$$

***Configuration associative strength value****:$V_{AB,0}^{\text{¢}}\left(2\right)=V_{A,1}(2)+V_{B,0}(2)+V_{AB,0}^{q}(2)=0$.*

**3^rd^ time unit**

***Stimulus presence*:** Both A and B are present in time-step 3. Following the CSC convention then, the components of both A, and B that were active at time-step 2 are now inactive. The next components in sequence then become active, i.e. $X_{A,3}\left(3\right)=1$, and $X_{B,2}\left(3\right)=1$.

***Configuration presence***: Applying Equation 4 as before, gives:$X_{AB}^{\text{¢}}\left(3\right)=\prod_{a} \sum_{b} X_{a,b}\left(3\right)-\text{min}(1,\sum_{i} \sum_{j} X_{i,j}\left(3\right))$. Since there are in this instance no other stimuli beyond those in the configuration, the second term becomes 0, hence:

$$X_{AB}^{\text{¢}}\left(3\right)=\left(X_{A,0}\left(3\right)+X_{A,1}\left(3\right)+X_{A,2}\left(3\right)\right)\left(X_{B,0}\left(3\right)+X_{B,1}\left(3\right)\right)-0=\left(0+0+1\right)\left(0+1\right)-0=1$$

This allows the calculation of Equation 5, which determines if the first component of the configuration is active:$X_{AB,0}^{\text{¢}}\left(3\right)=\max\left[0,X_{AB}^{\text{¢}}\left(3\right)-X_{AB}^{\text{¢}}\left(2\right)\right]=\max\left[0,0-1\right]=0$

Since from the previous time-step $X_{AB,0}^{\text{¢}}\left(2\right)=1$, we now calculate following Equation 6, thus $X_{AB,1}^{\text{¢}}\left(3\right)=X_{AB}^{\text{¢}}\left(3\right)X_{AB,0}^{\text{¢}}\left(2\right)=1\times1=1$ , i.e. the second component of the AB configuration (¢AB) identified as $X_{AB,1}^{\text{¢}}\left(3\right)$of is now active.

***Configural cue presence****:* Following Equation 7, the configural cue presence$X_{AB,1}^{q}\left(3\right)=1$ is instantiated.

***Stimulus associative strength value***: Obviously, the same parameters are used throughout the example ($\gamma=0.97$;$\rho=0.97$;$\beta=0.5$ $\alpha_{A}=0.2; \alpha_{B}=0.2; \alpha_{q_{AB}}=0.04$).

Turning our attention to the eligibility traces for the components of A, B and q_AB_:

$$e_{A,0}\left(3\right)=\min\left[1,0.97\times0.97\times1+0\right]=0.941$$

$$e_{A,1}\left(3\right)=\min\left[1,0.97\times0.97\times0+1\right]=1$$

$$e_{B,0}\left(3\right)=\min\left[1,0.97\times0.97\times0+1\right]=1$$

$$e_{AB,0}^{q}\left(3\right)=\min\left[1,0.97\times0.97\times0+1\right]=1$$

Finally, any change in associative strength can be calculated. Since no reinforcer has been presented on this time-step, associative strength for all components remains at 0.

**4^th^ time unit**

***Stimulus presence***: On this time-step, all stimuli cease, making all components inactive, and the reinforcer is delivered.

***Configuration presence***: This also applies to the configuration, as can be seen from Equation 4:

$X_{AB}^{\text{¢}}\left(4\right)=\left(X_{A,0}\left(4\right)+X_{A,1}\left(4\right)+X_{A,2}\left(4\right)\right)\left(X_{B,0}\left(4\right)+X_{B,1}\left(4\right)\right)-0=\left(0+0+0\right)\left(0+0\right)-0=0$.

Equation 5 remains zero: $X_{AB,0}^{\text{¢}}\left(4\right)=\max\left[0,X_{AB}^{\text{¢}}\left(4\right)-X_{AB}^{\text{¢}}\left(3\right)\right]=\max\left[0,0-1\right]=0$.

In addition, the presence of the second component of the AB configuration also becomes 0 following Equation 6: $X_{AB,1}^{\text{¢}}\left(4\right)=X_{AB}^{\text{¢}}\left(4\right)X_{AB,0}^{\text{¢}}\left(3\right)=0\times0=0$.

***Configural cue presence****:* It then follows that $X_{AB,0}^{q}\left(4\right)$ and $X_{AB,1}^{q}\left(4\right)$ are also zero.

The eligibility traces of the three stimuli compute as follows:

$$e_{A,0}\left(4\right)=\min\left[1,0.97\times0.97\times0.941+0\right]=0.885$$

$$e_{A,1}\left(4\right)=\min\left[1,0.97\times0.97\times1+0\right]=0.941$$

$$e_{A,2}\left(4\right)=\min\left[1,0.97\times0.97\times0+1\right]=1$$

$$e_{B,0}\left(4\right)=\min\left[1,0.97\times0.97\times1+0\right]=0.941$$

$$e_{B,1}\left(4\right)=\min\left[1,0.97\times0.97\times0+1\right]=1$$

$$e_{AB,0}^{q}\left(4\right)=\min\left[1,0.97\times0.97\times1+0\right]=0.941$$

$$e_{AB,1}^{q}\left(4\right)=\min\left[1,0.97\times0.97\times0+1\right]=1$$

The introduction of the US on this time-step necessitates evaluating Equation 1, to determine $\delta V$ for the components. Using the parameters defined earlier,$\delta V_{i,j}\left(4\right)=X_{0}\left(4\right)+\gamma\left[\sum_{i} \sum_{j} X_{i,j}\left(4\right)V_{i,j}\left(3\right)\right]-\left[\sum_{i} \sum_{j} X_{i,j}\left(4\right)V_{i,j}\left(4\right)\right]=1$

Note that at this first delivery of the US, the prediction error is 1 because no component has associative strength, and hence both summation terms are zero.

Taking this forward to Equation 2, the new associative strength of the components can be calculated as follows:

$$V_{A,0}\left(4\right)=V_{A,0}\left(3\right)+\beta\alpha_{A}\delta V_{i,j}\left(4\right)e_{A,0}\left(4\right)=0+0.5\times0.2\times1\times0.885=0.089$$

$$V_{A,1}\left(4\right)=V_{A,1}\left(3\right)+\beta\alpha_{A}\delta V_{i,j}\left(4\right)e_{A,1}\left(4\right)=0+0.5\times0.2\times1\times0.941=0.094$$

$$V_{A,2}\left(4\right)=V_{A,2}\left(3\right)+\beta\alpha_{A}\delta V_{i,j}\left(4\right)e_{A,2}\left(4\right)=0+0.5\times0.2\times1\times1=0.1$$

And similarly for stimulus B:

$$V_{B,0}\left(4\right)=V_{B,0}\left(3\right)+\beta\alpha_{B}\delta V_{i,j}\left(4\right)e_{B,0}\left(4\right)=0+0.5\times0.2\times1\times0.941=0.094$$

$$V_{B,1}\left(4\right)=V_{B,1}\left(3\right)+\beta\alpha_{B}\delta V_{i,j}\left(4\right)e_{B,1}\left(4\right)=0+0.5\times0.2\times1\times1=0.1$$

And for the configural cue, q_AB_:

$$V_{AB,0}^{q}\left(4\right)=V_{AB,0}^{q}\left(3\right)+\beta\alpha_{AB}^{q}\delta V_{i,j}\left(4\right)e_{AB,0}^{q}\left(4\right)=0+0.5\times0.04\times1\times0.941=0.0188$$

$$V_{AB,1}^{q}\left(4\right)=V_{AB,1}^{q}\left(3\right)+\beta\alpha_{AB}^{q}\delta V_{i,j}\left(4\right)e_{AB,1}^{q}\left(4\right)=0+0.5\times0.04\times1\times1=0.02$$

***Configuration associative strength value****:*$V_{AB,0}^{\text{¢}}\left(4\right)=V_{A,1}(4)+V_{B,0}(4)+V_{AB,0}^{q}(4)=0.207$ and $V_{AB,1}^{\text{¢}}\left(4\right)=V_{A,2}(4)+V_{B,1}(4)+V_{AB,1}^{q}(4)=0.22$

N.B. These values are those given by the simulator for each stimulus component at trial 1.

ITI**:**

During the ITI, no stimuli are present and the eligibility traces for all three CSs gradually decline.

Trial 2**:**

**6^th^ time unit**

***Stimulus presence***: Trial 2 proceeds similarly to trial 1, with A present on the first step making$X_{A,0}(6)=1$ and$X_{B,0}(6)=0$.

***Configuration presence***: As in the first trial, Equations 4, 5, and 6 evaluate to zero at this time-step, because the absence of B implies that the ¢AB configuration must also be absent.

***Configural cue presence***: Since the configuration is not present, no configural cue is assumed, thus$X_{AB,0}^{q}\left(6\right)=0$.

***Stimulus associative strength value***: On this trial, the stimuli have begun to acquire associative strength. The first component of A is present, with an associative strength $V_{A,0}\left(6\right)=0.089$, the value obtained at $V_{A,0}\left(4\right)$, weakly predicting the US. At this time-step, the eligibility traces of all stimuli are at zero and hence no change to associative strength can occur.

**7^th^ time unit**

***Stimulus presence***: As before, the first component of A becomes inactive, and the second is now active as is the first component of B:$X_{A,0}(7)=0$, $X_{A,1}(7)=1$and$X_{B,0}(7)=1$.

***Configuration presence***: As in the first trial, Equations 4, and 5 now yield $X_{AB}^{\text{¢}}(7)=1$ and $X_{AB,0}^{\text{¢}}\left(7\right)=1$

***Configural cue presence*:** The first component of the AB configuration is now present, leading to$X_{AB,0}^{q}\left(7\right)=1$.

***Stimulus associative strength value***: At this time-step, there is a notable change in the TD error:

$$\delta V_{i,j}\left(7\right)=X_{0}\left(7\right)+\gamma\left[\sum_{i} \sum_{j} X_{i,j}\left(7\right)V_{i,j}\left(6\right)\right]-\left[\sum_{i} \sum_{j} X_{i,j}\left(6\right)V_{i,j}\left(6\right)\right]$$

$$\begin{matrix} \delta V_{A,0}\left(7\right)=X_{0}\left(7\right)+ \gamma\left[\begin{matrix} \left(X_{A,0}\left(7\right)\times V_{A,0}\left(6\right)\right)+\left(X_{A,1}\left(7\right)\times V_{A,1}\left(6\right)\right)+\left(X_{B,0}\left(7\right)\times V_{B,0}\left(6\right)\right)+ \\ \left(X_{B,1}\left(7\right)\times V_{B,1}\left(6\right)\right)+\left(X_{AB,0}^{q}\left(7\right)\times V_{AB,0}^{q}\left(6\right)\right)+\left(X_{AB,1}^{q}\left(7\right)\times V_{AB,1}^{q}\left(6\right)\right) \end{matrix}\right] \\ -\left[\begin{matrix} \left(X_{A,0}\left(6\right)\times V_{A,0}\left(6\right)\right)+\left(X_{A,1}\left(6\right)\times V_{A,1}\left(6\right)\right)+\left(X_{B,0}\left(6\right)\times V_{B,0}\left(6\right)\right)+ \\ \left(X_{B,1}\left(6\right)\times V_{B,1}\left(6\right)\right)+\left(X_{AB,0}^{q}\left(6\right)\times V_{AB,0}^{q}\left(6\right)\right)+\left(X_{AB,1}^{q}\left(6\right)\times V_{AB,1}^{q}\left(6\right)\right) \end{matrix}\right] \end{matrix}$$

Substituting,

$$\begin{matrix} \delta V_{i,j}\left(7\right)=0+ 0.97\left[0\times0.089+1\times0.094+0\times0.1+1\times0.094+0\times0.1+1\times0.0188+0\times0.02\right] \\ -\left[1\times0.089+0\times0.094+0\times0.1+0\times0.094+0\times0.1+0\times0.0188+0\times0.02\right] \\ =0.97\left[0.094+0.094+0.0188\right]-0.089=0.207\times0.97-0.089=0.112 \end{matrix}$$

Combining with the eligibility traces, which are zero for all components but the first of A, $e_{A,0}\left(7\right)=\text{min}\left[1, 0.97\times0.97\times0+1\right]=1$, entails that the associative strength of the first component of A increases, while the others remain unchanged:

$$V_{A,0}\left(7\right)=V_{A,0}\left(6\right)+\beta\alpha_{A}\delta V_{i,j}\left(7\right)e_{A,0}\left(7\right)=0.089+0.5\times0.2\times0.112\times1=0.100$$

$$V_{A,1}(7)=0.94; V_{B,0}(7)=0.94; V_{AB,0}^{q}\left(7\right)=0.0188; V_{AB,0}^{\text{¢}}=0.207$$

**8^th^ time unit**

***Stimulus presence***: On this time-step the final components of the stimuli are active, and all others inactive:$X_{A,0}(8)=0; X_{A,1}(8)=0; X_{A,2}(8)=1; X_{B,0}(8)=0; X_{B,1}(8)=1$.

***Configuration presence***: The overall configuration remains present, but the first component is now inactive:$X_{AB}^{\text{¢}}(8)=1; X_{AB,0}^{\text{¢}}(8)=0$. Equation 6 now shows that the second component is active:

$X_{AB,1}^{\text{¢}}\left(8\right)=X_{AB}^{\text{¢}}\left(8\right)X_{AB,0}^{\text{¢}}\left(7\right)=1\times1=1$.

***Configural cue presence***: Naturally, this implies that$X_{AB,0}^{q}\left(8\right)=0; X_{AB,1}^{q}\left(8\right)=1$.

***Stimulus associative strength value***: Evaluating Equation 1 shows that the TD error is once again positive on this time-step:

$$\delta V_{i,j}\left(8\right)=X_{0}\left(8\right)+\gamma\left[\sum_{i} \sum_{j} X_{i,j}\left(8\right)V_{i,j}\left(7\right)\right]-\left[\sum_{i} \sum_{j} X_{i,j}\left(7\right)V_{i,j}\left(7\right)\right]$$

$$\begin{matrix} \delta V_{i,j}\left(8\right)=0+ \gamma\left[0\times0.100+0\times0.094+1\times0.1+0\times0.094+1\times0.1+0\times0.0188+1\times0.02\right] \\ -\left[0\times0.100+1\times0.094+0\times0.1+1\times0.094+0\times0.1+1\times0.0188+0\times0.02\right] \\ =\gamma\left[0.1+0.1+0.02\right]-\left[0.094+0.094+0.0188\right]=0.22\gamma-0.207=0.007 \end{matrix}$$

With eligibility traces $e_{A,0}\left(8\right)=0.941$, and $e_{A,1}\left(8\right)=e_{B,0}\left(8\right)=e_{AB,0}^{q}\left(8\right)=1$:

$$V_{A,0}\left(8\right)=V_{A,0}\left(7\right)+\beta\alpha_{A}\delta V_{i,j}\left(8\right)e_{A,0}\left(8\right)=0.100+0.5\times0.2\times0.007\times0.941=0.101$$

$$V_{A,1}\left(8\right)=V_{A,1}\left(7\right)+\beta\alpha_{A}\delta V_{i,j}\left(8\right)e_{A,1}\left(8\right)=0.094+0.5\times0.2\times0.007\times1=0.0947$$

$$V_{A,2}\left(8\right)=V_{A,1}\left(7\right)+\beta\alpha_{A}\delta V_{i,j}\left(8\right)e_{A,2}\left(8\right)=0.1+0=0.1$$

$$V_{B,0}\left(8\right)=V_{B,0}\left(7\right)+\beta\alpha_{B}\delta V_{i,j}\left(8\right)e_{B,0}\left(8\right)=0.094+0.5\times0.2\times0.007\times1=0.0947$$

$$V_{B,1}\left(8\right)=V_{B,1}\left(7\right)+\beta\alpha_{B}\delta V_{i,j}\left(8\right)e_{B,1}\left(8\right)=0.1+0=0.1$$

$$V_{AB,0}^{q}\left(8\right)=V_{AB,0}^{q}\left(7\right)+\beta\alpha_{AB}^{q}\delta V_{i,j}\left(8\right)e_{AB,0}^{q}\left(8\right)=0.0188+0.5\times0.04\times0.007\times1=0.0189$$

$$V_{AB,1}^{q}\left(8\right)=V_{AB,1}^{q}\left(7\right)+\beta\alpha_{AB}^{q}\delta V_{i,j}\left(8\right)e_{AB,1}^{q}\left(8\right)=0.0188+0=0.0189$$

***Configuration associative strength value****:*

$V_{AB,0}^{\text{¢}}\left(8\right)=V_{A,1}(8)+V_{B,0}(8)+V_{AB,0}^{q}(8)=0.208$ and $V_{AB,1}^{\text{¢}}\left(8\right)=V_{A,2}(8)+V_{B,1}(8)+V_{AB,1}^{q}(8)=0.1+0.1+0.0189=.0219$

**9^th^ time unit**

Following the same procedure as above will get:

$$\begin{matrix} \delta V_{i,j}\left(9\right)=1-\left[0\times0.101+0\times0.0947+1\times0.1+0\times0.094+1\times0.1+0\times0.0188+1\times0.02\right] \\ =1-\left[0.1+0.1+0.02\right]=1-0.22=0.78 \end{matrix}$$

$$e_{A,0}\left(9\right)=\min\left[1,0.97\times0.97\times0.941+0\right]=0.885$$

$$V_{A,0}\left(9\right)=V_{A,0}\left(8\right)+\beta\alpha_{A}\delta V_{i,j}\left(9\right)e_{A,0}\left(9\right)=0.101+0.5\times0.2\times0.78\times0.885=0.17$$

and the same for the remaining elements.

Thus, the final results at the end of the trial will be

$V_{A,0}\left(9\right)=0.17$ ; $V_{A,1}\left(9\right)=0.169$ ; $V_{A,2}\left(9\right)=.0178$

$V_{B,0}\left(9\right)=0.168$; $V_{B,1}\left(9\right)=0.178$

$V_{AB,0}^{q}\left(9\right)=0.034$ ; $V_{AB,1}^{q}\left(9\right)=0.036$

$V_{AB,0}^{\text{¢}}\left(9\right)=0.37$; $V_{AB,1}^{\text{¢}}\left(9\right)=0.392$

This terminates the step by step illustration of how the equations of the SSCC TD model compute.

Please notice that the values above may vary slightly from those output by the SSCC TD Simulator because they were rounded to the 3^rd^ decimal. Also notice that the simulated values per stimulus and component that are obtained with the simulator correspond to the final component values at the end of each trial.

| **Symbols and parameters** | **Values** | **Denotation** |
| --- | --- | --- |
| $\gamma$ | $0\leq\gamma\leq1$ | Discount factor |
| $\rho$ | $0\leq\rho\leq1$ | Decay parameter |
| $\alpha$ | $0\leq\alpha\leq1$ | Learning rate, CS salience |
| $\beta$ | $0\leq\beta\leq1$ | US salience. Different values can be given to reinforced ($\beta+$) and non-reinforced ($\beta-$) trials |
| $\lambda$ ($X_{0}$ in equations) | $0\leq\lambda\leq1$ | US asymptotic level |
| A, B, …, Z |  | Stimuli. Combinations of these represent compound *m*. In a given compound they are called primitive constituents |
| $q_{m}$ |  | Configural cue corresponding to compound *m* |
|  |  | Configuration consisting of primitive constituents (e.g., A and B) and configural cue (e.g., $q_{AB}$) |
| $e_{i,j}$ | $0\leq e\leq1$ | Eligibility trace of component *j* of stimulus *i* |
| $X_{i,j}$ | 0 or 1 | Presence (1) or absence (0) of component *j* of stimulus *i* |
| $X_{a,b}$ | 0 or 1 | Presence (1) or absence (0) of component *b* of stimulus *a* in *target* compound |
| $X_{m,n}^{\text{¢}}$ | 0 or 1 | Presence (1) or absence (0) of component *n* of simultaneous compound *m* |
| $X_{m',n}^{\text{¢}}$ | 0 or 1 | Presence (1) or absence (0) of component *n* of serial compound *m’* |
| $X_{m.n}^{q}$ | 0 or 1 | Presence (1) or absence (0) of component *n* of configural cue *m* |
| $Y_{k,l}$ | 0 or 1 | Presence (1) or absence (0) of component *l* of the trace stimulus *k* |
| $V$ |  | Associative strength |
| $\delta V$ |  | Update rule (aka TD error) |
|  |  | duration of the context in trial $T$ |
| $B$ | $0\leq B\leq1$ | Decision rule threshold |

**Table S1.** Glossary of symbols and parameters, range values (where appropriate), and denotation.
