# Supplementary material for: SSCC TD: A Serial and Simultaneous Configural-Cue Compound Stimuli Representation for Temporal Difference Learning
Source: PLoS One. 2014 Jul 23;9(7):e102469. doi: 10.1371/journal.pone.0102469 (PMC4108321; doi:10.1371/journal.pone.0102469)
Supplement: Simulator Quick Guide S1 — A nutshell guide to use the SSCC TD Simulator. (DOCX) [file pone.0102469.s003.docx]

**Quick step-by-step guide to run the simulations**

1. Download a version of the simulator appropriate to your platform from <http://www.cal-r.org/index.php?id=SSCC_TD_sim>.
2. For the simulator to run you need to have Java installed in your machine. ^[[1]](#footnote-1)^
3. Download the .scc files of the experiments you want to simulate from the Supporting Information files at the journal repository.
4. After launching the simulator select the “Open” function from the “File” menu and choose the experiment you wish to run, for instance, “Exp2_Blocking_AP&G_02.ssc”
5. This will retrieve the design of the experiment in the main group/phase grid.
6. Press “Set Parameters” to recover all the settings (configural cues, etc.) needed to run the experiment.
7. Click the “Run” button at the bottom left.
8. Once the simulation has been completed (be aware that it can take a long time in some cases) export the results choosing the corresponding function from the “File” menu. This creates a ‘.xlsx’ spreadsheet file on the selected directory. The workbook will open a sheet per group, headed by the name of the file, followed by the parameters. Experimental Phases are presented individually on separate tables. Each Phase table shows the V (and Responses) results per CSC for each trial, as well as the mean V (and Responses) for the whole stimulus (or configuration) on each trial.
9. If you wish to visualize the results graphically, click the “Display Figures” button.

Of course, you can download the spreadsheets for the experiments in the paper directly from the Supporting Information files at the journal repository.

For a more comprehensive guide users are referred to

Gray, J., Mondragón, E. & Alonso, E. (2013). SSCC_TD Simulator v.1. *CAL Simulation Guides ISSN 2054-2747*. Retrieved from <http://www.cal-r.org/index.php?id=CAL-SIM-Guides>.

1. To run any of these files you will need to have the Java Runtime Environment (JRE) 6 or above installed in your machine. Apple and Linux distributions already include a JRE. Many Windows applications also require JRE so chances are that you have it already installed; if not, you can download it free from Oracle.com. [↑](#footnote-ref-1)
